# Uridine inhibits ROS-mediated osteoclast differentiation and alleviates osteoporosis via modulation of PI3K/Akt–FoxO signaling

**DOI:** 10.3389/fimmu.2026.1767279

**Published:** 2026-04-27

**Authors:** Sijie Bian, Lianhui Zhao, Xu Wang, Zhangwei Wu, Maolin Yang, Jianliang Ou, Tao Han, Faxue Liao, Qingkai Xue, Xingxing Huo, Jun Chang

**Affiliations:** 1 Department of Orthopaedics, The First Affiliated Hospital of Anhui Medical University, Hefei, China; 2 Anhui Public Health Clinical Center, Hefei, China; 3 School of Basic Medical Sciences, Anhui Medical University, Hefei, China; 4 Experimental Center of Clinical Research, Scientific Research Department, The First Affiliated Hospital of Anhui University of Chinese Medicine, Hefei, Anhui, China

**Keywords:** multi-omics, osteoclast differentiation, osteoporosis, PI3K/Akt–FoxO, uridine

## Abstract

**Background:**

Osteoporosis is a metabolic bone disease characterized by dysregulated osteoclast activity, resulting in increased bone degradation and compromised bone microarchitecture. While the interconnection between osteoclast differentiation and cellular energy metabolism has become increasingly recognized, the role of pyrimidine metabolism in this process remains largely undefined.

**Methods:**

Integrative multi-omics analyses were performed to characterize transcriptional and metabolic alterations during receptor activator of nuclear factor-κB ligand (RANKL)-induced osteoclast differentiation. The effects of uridine (UD) on osteoclast development and resorptive function were assessed in vitro using RAW264.7 cells and bone marrow-derived macrophages (BMMs). In vivo effects of UD on bone loss were evaluated in an ovariectomized (OVX) mouse model.

**Results:**

Integrative analyses revealed distinct metabolic remodeling during osteoclast differentiation and identified UD as a pivotal metabolite that showed a significant decline upon RANKL stimulation. Experimental evidence indicated that exogenous UD supplementation significantly suppressed osteoclast development and resorptive function, along with a reduction in the expression of nuclear factor of activated T cells c1 (NFATc1) and cathepsin K (CTSK). In OVX mice, UD administration improved trabecular microarchitecture, reduced osteoclast burden, and mitigated bone loss. Mechanistically, UD inhibited phosphoinositide 3-kinase/protein kinase B (PI3K/Akt) phosphorylation, facilitated Forkhead box O (FoxO) nuclear translocation, and suppressed reactive oxygen species (ROS) accumulation, thereby preventing NFATc1 activation and nuclear import.

**Conclusion:**

Collectively, this research identifies a novel metabolic–signaling interplay linking pyrimidine metabolism with osteoclast differentiation and highlights UD as a promising metabolic regulator for the treatment and prevention of osteoporosis.

## 1 Introduction

Osteoporosis is a systemic skeletal disorder characterized by a progressive reduction in bone mass and disruption of bone microarchitecture, ultimately increasing the risk of fractures (1, 2). With the rapid aging of the global population, the disease has become a major public health challenge worldwide (3, 4). Epidemiological studies estimate that over 200 million people are affected globally (5), with the incidence being significantly higher in women, primarily due to estrogen deficiency (6, 7). Currently, most therapeutic approaches focus on inhibiting bone resorption, primarily through the use of bisphosphonates and monoclonal antibodies targeting the receptor activator of nuclear factor-κB ligand (RANKL) (8, 9). However, the long-term efficacy of these treatments is often limited by adverse effects and rapid bone loss following treatment discontinuation (10). Therefore, elucidating the novel molecular mechanisms and identifying safe and durable pharmacological interventions remain critical priorities for the prevention and management of osteoporosis.

Osteoporosis is characterized by excessive bone resorption driven by overactivated osteoclasts (6, 11). Osteoclast differentiation is primarily regulated through RANKL signaling and its downstream transcription factor, nuclear factor of activated T cells c1 (NFATc1) (12), which orchestrates the expression of osteoclast-specific genes (13). Increasing evidence suggests that reactive oxygen species (ROS) play a key role in this process, as their excessive accumulation enhances osteoclast formation and bone resorptive activity (14–16). Acting as both effectors and metabolic indicators, ROS serve as a critical link between cellular metabolism and osteoclast differentiation by reflecting mitochondrial function and redox homeostasis (17, 18). At the signaling level, intracellular ROS homeostasis is dynamically regulated by redox-sensitive pathways. Among these, Forkhead box O (FoxO) transcription factors are key downstream effectors of the phosphoinositide 3-kinase (PI3K)/protein kinase B (Akt) pathway and play a central role in coordinating oxidative stress responses, cellular metabolism, and survival (19). Activation of PI3K/Akt signaling leads to FoxO phosphorylation and subsequent nuclear exclusion, thereby suppressing FoxO-dependent transcription of antioxidant and stress-responsive genes, such as those involved in ROS detoxification. Conversely, inhibition of PI3K/Akt signaling restores FoxO nuclear activity and promotes antioxidant gene expression, contributing to redox homeostasis (20, 21). Given the critical role of ROS in driving osteoclastogenesis, dysregulation of the PI3K/Akt–FoxO axis may therefore facilitate excessive ROS accumulation and aberrant osteoclast differentiation. However, most studies to date have focused on glucose and lipid metabolism (22, 23), whereas the role of nucleotide metabolism, particularly pyrimidine metabolism, in redox regulation and osteoclast differentiation remains largely unexplored.

Pyrimidine metabolism is a fundamental metabolic pathway that sustains cellular homeostasis by supporting nucleotide synthesis, energy supply, and signal regulation, making it essential for cell growth and differentiation (24, 25). Previous studies have shown that uridine (UD) exerts antioxidant and cytoprotective effects in neurological and metabolic disorders by modulating mitochondrial activity and oxidative stress responses, thereby maintaining intracellular energy balance (26–28). Accumulating evidence indicates that pyrimidine metabolism is significantly dysregulated in osteoporosis, as evidenced by reduced levels of UD and related metabolites in bone tissue. Furthermore, lower circulating UD concentrations are closely associated with an increased risk of osteoporosis (29–31). Despite these findings, the functional significance and regulatory mechanisms of UD in bone metabolism remain largely unexplored.

This study integrated transcriptomic and metabolomic analyses to identify the key metabolic changes during osteoclast differentiation. The results revealed a significant reduction in UD, one of the main metabolites involved in the pyrimidine metabolic pathway. Subsequent *in vitro* and *in vivo* investigations demonstrated that supplementation with exogenous UD effectively suppressed osteoclast differentiation and alleviated osteoporotic symptoms in mice. Mechanistically, UD inhibited osteoclast differentiation by promoting the elimination of ROS through modulation of the PI3K/Akt–FoxO signaling pathway. This work provides new insights into the metabolic mechanisms underlying osteoporosis and highlights potential therapeutic approaches.

## 2 Materials and methods

### 2.1 Chemicals and reagents

Recombinant mouse macrophage colony-stimulating factor (M-CSF) (51112-MNAH) and RANKL (462-TEC) were provided by Sino Biological (Beijing, China) and R&D Systems (Minneapolis, MN, USA), respectively. UD (HY-B1449), LY294002 (HY-10108), 740Y-P (HY-P0175), lipopolysaccharide (LPS) (HY-D1056), N-acetylcysteine (NAC) (HY-B0215), and alendronate sodium (HY-11101) were purchased from MedChemExpress (Shanghai, China). Hoechst 33342 (C1026), Actin-Tracker (C2205S), the nuclear and cytoplasmic protein extraction kit (P0028), and the DCFH-DA ROS detection kit (S0034S) were sourced from Beyotime (Shanghai, China). The cell counting kit-8 (CCK-8) detection kit (A311-01) was obtained from Vazyme (Nanjing, China), and the tartrate-resistant acid phosphatase (TRAP) staining kit (BB-4421) was supplied by Shanghai Beibokit Biotechnology Co., Ltd; (Shanghai, China). Primary antibodies against NFATc1 (ab25916), cathepsin K (CTSK) (ab187647), FoxO1 (18592-1-AP), and p-FoxO1 (Thr24, #9464) were acquired from Abcam (Cambridge, UK) and Proteintech (Wuhan, China). Antibodies for PI3K (60225-1-Ig), p-PI3K (Tyr458, #17366), Akt (60203-2-Ig), and p-Akt (Ser473, 80455-1-RR) were sourced from Cell Signaling Technology (USA) and Immunoway (USA). GAPDH (YM8394), Lamin B1 (66095-1-Ig), and Tubulin (YM8514) were provided by Proteintech (Wuhan, China). Horseradish peroxidase (HRP)-conjugated secondary antibodies were purchased from ZSGB-Bio (Beijing, China). Dulbecco’s modified Eagle medium (DMEM) (C11330500BT), alpha minimum essential medium (α-MEM) (C12571500BT), fetal bovine serum (FBS) (BC-SE-FBS01), and penicillin–streptomycin (15140122) were obtained from Gibco (Thermo Fisher Scientific, USA).

### 2.2 Cell culture and osteoclast differentiation

The RAW264.7 murine macrophage cell line was purchased from the Cell Bank of the Chinese Academy of Sciences (Shanghai, China). Primary bone marrow–derived macrophages (BMMs) were isolated from the femoral and tibial bones of 6- to 8-week-old female C57BL/6 mice. Both cell types were cultured at 37 °C with 5% CO_2_ in a humidified incubator, in complete culture medium based on either DMEM or α-MEM, supplemented with 10% FBS and 1% penicillin–streptomycin. For BMM preparation, bone marrow cells were rinsed and centrifuged with α-MEM. After 24 hours of culture, adherent cells were removed, and the non-adherent cells in suspension were collected and stimulated with M-CSF (40 ng/mL) to induce differentiation into purified BMMs.

For osteoclast differentiation, RAW264.7 cells (32) or BMMs (33) were stimulated with RANKL (50 ng/mL) for 3–5 days, with medium refreshed every 2 days. UD or pathway modulators were added as indicated. For downstream molecular analyses, cells were harvested 72 h after RANKL stimulation, unless otherwise specified.

### 2.3 Determination of cell viability and UD content

Cell viability was assessed using a CCK-8 assay kit. After 48 hours of treatment, 10 μL of detection reagent was added to each well and incubated at 37 °C for 2 hours. Absorbance at 450 nm was then measured to evaluate cell viability.

Intracellular UD levels were measured in cell lysates by enzyme-linked immunosorbent assay (ELISA). Cell lysates from different treatments were added to antibody-coated wells, followed by incubation with detection reagents. The absorbance at 450 nm was recorded, and UD concentrations were calculated based on the standard curve.

### 2.4 Osteoclast differentiation and functional evaluation

Cells were fixed with 4% paraformaldehyde for 15 minutes and then subjected to TRAP staining. Multinucleated TRAP-positive cells (≥3 nuclei) were identified as osteoclasts. For F-actin staining, cells were treated with 0.1% Triton X-100 for membrane permeabilization, blocked with 5% BSA, and labeled with tetramethylrhodamine isothiocyanate (TRITC)-phalloidin and Hoechst. Actin rings were visualized by confocal microscopy and quantified using ImageJ. Bone resorption was assessed on bovine bone slices cultured with RANKL ± UD for 7–9 days, followed by rinsing, drying, and analysis of pit area using ImageJ.

### 2.5 Quantitative RT-PCR analysis

Total RNA was isolated using the RNA-easy Isolation Reagent. Complementary DNA (cDNA) was synthesized by reverse transcription, and quantitative real-time PCR was performed using a SYBR Green Master Mix. GAPDH was used as the internal reference, and relative gene expression levels were calculated using the 2^-ΔΔCt^ method.

### 2.6 Western blot analysis

Proteins were extracted from cells and bone tissues using radioimmunoprecipitation assay buffer (RIPA) buffer containing protease and phosphatase inhibitors. Nuclear and cytoplasmic fractions were separated when necessary. Tissues were pulverized in liquid nitrogen and lysed on ice. Equal amounts of protein were resolved by sodium dodecyl sulfate polyacrylamide gel electrophoresis (SDS-PAGE) and transferred to membranes. After blocking for 30 minutes at room temperature, membranes were incubated overnight at 4 °C with primary antibodies. HRP-conjugated secondary antibodies were applied the following day. Protein bands were visualized using enhanced chemiluminescence (ECL) and analyzed with ImageJ.

### 2.7 Immunofluorescence and ROS detection

After fixation and permeabilization, cells were blocked with 5% BSA and then incubated overnight at 4 °C with primary antibodies targeting NFATc1 or FoxO. The following day, cells were incubated with fluorescent secondary antibodies and Hoechst for staining. Protein localization was observed using a confocal microscope.

For ROS detection, cells were incubated with 10 μM DCFH-DA for 30 minutes, rinsed with PBS, and visualized using a fluorescence microscope. Fluorescence intensity was quantified using ImageJ.

### 2.8 Animal models and grouping procedures

Female C57BL/6 mice, aged eight weeks (18–22 g), were supplied by Hefei Qingyuan Biotechnology Co., Ltd. The animals were housed under specific pathogen-free (SPF) conditions. All experimental procedures were reviewed and approved by the Institutional Animal Care and Use Committee of the First Affiliated Hospital, Anhui University of Chinese Medicine (Approval No. AZYFY-2025-2007).

Mice were randomly assigned to the different experimental groups at the beginning of the study. Osteoporosis was induced by bilateral ovariectomy (OVX) in all mice except those in the sham group (34). All surgical procedures were performed under general anesthesia induced by intraperitoneal injection of sodium pentobarbital (50 mg/kg). After a one-week recovery period, the animals were randomly assigned to five experimental groups: sham, OVX model, alendronate (ALN, 2 mg/kg) as the positive control, and UD treatment groups at high, medium, and low doses (200, 100, and 50 mg/kg) (35). UD doses were selected based on its endogenous nature and previously reported dosing ranges in the literature. The sham group underwent the same surgical procedure without ovary removal. All treatments were administered via daily oral gavage over an 8-week period. At the end of the experimental period, mice were euthanized by intraperitoneal injection of sodium pentobarbital (150 mg/kg), and femurs and tibias were collected for histological and biochemical analyses.

### 2.9 Bone morphology and histopathological analysis

Femurs were harvested and fixed in 4% paraformaldehyde for 48 hours. Bone microarchitecture was assessed using micro-computed tomography (micro-CT; Raycision, China). Measurements were taken at the distal femur, approximately 1 mm below the growth plate. 3D reconstruction and morphometric analysis were performed using CTAn software.

Decalcification of bone specimens was performed using a 10% EDTA solution (pH 7.4). The decalcified tissues were then paraffin-embedded and sectioned into 5 μm-thick slices. For histological examination, H&E staining was used to evaluate bone morphology, while TRAP staining was applied to visualize osteoclast distribution. Immunohistochemistry was carried out according to established procedures, and image data were quantified using the ImageJ analysis platform.

### 2.10 Transcriptomic and metabolomic analyses

RNA sequencing and metabolomic analyses were conducted by Tsingke Biotechnology (China). For RNA sequencing, total RNA was assessed for quality, and libraries were sequenced using the Illumina NovaSeq platform. Differentially expressed genes (DEGs) were identified using DESeq2, followed by gene ontology (GO), Kyoto encyclopedia of genes and genomes (KEGG), and gene set enrichment analysis (GSEA) enrichment analyses.

Metabolomic profiling was performed using a liquid chromatography–tandem mass spectrometry (LC–MS/MS) platform. After data normalization, principal component analysis (PCA) and orthogonal partial least squares discriminant analysis (OPLS-DA) were applied to assess metabolic differences between groups. KEGG enrichment and integrated transcriptome–metabolome analyses were used to identify key metabolic pathways and metabolite–gene interactions.

### 2.11 Statistical analysis

All experiments were performed at least in triplicate, and data are presented as the mean ± standard deviation (SD). For group comparisons, statistical differences were analyzed using one-way or two-way analysis of variance (ANOVA), followed by Tukey’s or Bonferroni multiple comparison tests. Data processing and analysis were conducted using GraphPad Prism software (version 10.1.2), with p-values less than 0.05 considered statistically significant.

## 3 Results

### 3.1 Pyrimidine metabolism was significantly remodeled during osteoclast differentiation

**Figure 1:**
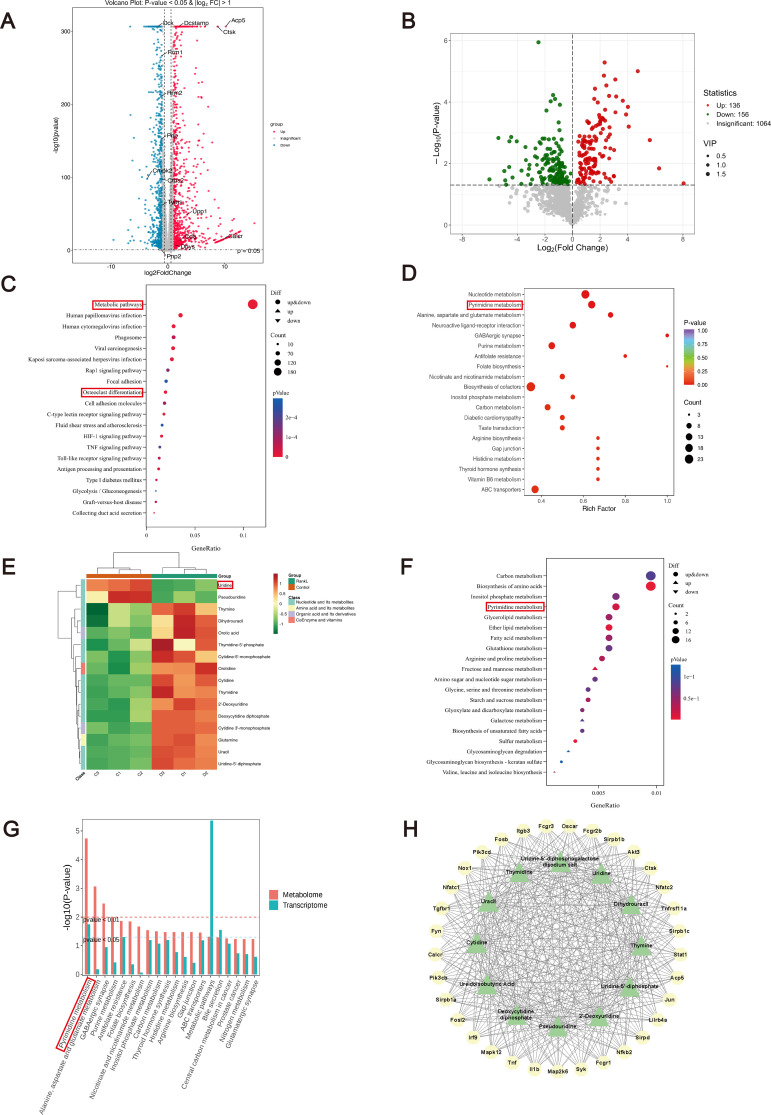
Transcriptomic and metabolomic analyses of RANKL-induced osteoclast differentiation. **(A, B)** Volcano plots of DEGs **(A)** and metabolites **(B)** between control and RANKL groups; **(C, D)** KEGG pathway enrichment of DEGs **(C)** and metabolites **(D, E)** Heatmap of metabolites significantly altered after RANKL stimulation; **(F)** KEGG enrichment of metabolism-related pathways from transcriptomic data; **(G)** Integrated KEGG analysis combining transcriptomic and metabolomic datasets; **(H)** Metabolite–gene interaction network.

We integrated metabolomic and transcriptomic analyses to delineate the metabolic reprogramming occurring during RANKL-driven osteoclast differentiation, a key process in osteoporosis. Metabolomic profiling revealed significant alterations in cellular metabolism following induction, with PCA confirming a clear separation between the control and RANKL-induced groups (**Supplementary Figures 1A, B**). Differential metabolite analysis (**Figure 1B**) identified 136 metabolites that were significantly upregulated and 156 metabolites that were significantly downregulated. KEGG enrichment analysis highlighted pyrimidine metabolism as a key pathway in both omics layers (**Figures 1C–G**). Heatmaps underscored changes in pyrimidine-related metabolites, with UD showing a significant decrease (**Figure 1E**). The metabolite–gene interaction network (**Figure 1H**) demonstrated strong associations between pyrimidine metabolites and osteoclast-related genes.

**Figure 2:**
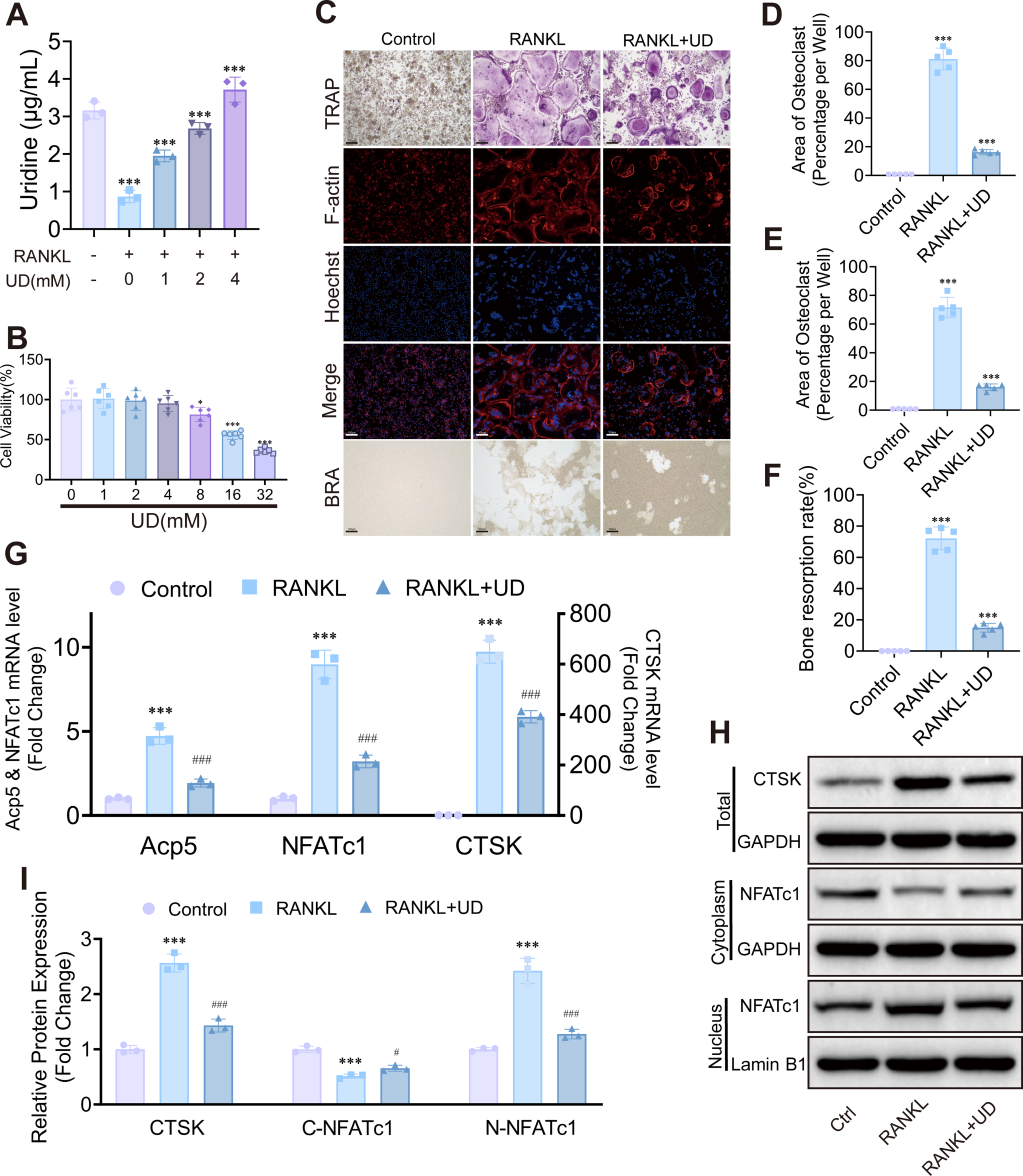
Uridine inhibited osteoclast differentiation and bone resorption. **(A)** Intracellular uridine levels; **(B)** Cell viability following 48 h uridine exposure; **(C–F**) TRAP, F-actin ring, and bone-resorption assays with corresponding quantitative analyses, Scale bar = 100 μm; **(G)** Relative mRNA expression of *CTSK*, *NFATc1*, and *Acp5*; **(H, I)** Western blot and densitometric analysis of CTSK and cytoplasmic/nuclear NFATc1. Data represent mean ± SD (n = 3). *p* < 0.05, *p < 0.01, **p < 0.001 vs Control; #*p* < 0.05, ##*p* < 0.01, ###*p* < 0.001 vs RANKL.

Based on these findings, we focused on key metabolites involved in pyrimidine metabolism. Among these metabolites, uridine was selected for subsequent investigation. Quantitative analysis based on ELISA showed an obvious reduction in intracellular UD levels during osteoclast differentiation (**Figure 2A**). These results suggest that UD metabolism may play an important regulatory role in osteoclast differentiation, and that exogenous UD supplementation may modulate this process.

### 3.2 UD inhibited osteoclast differentiation and bone resorption *in vitro*

To examine the effect of intracellular UD levels on osteoclast differentiation, primary BMMs and RAW264.7 cells were differentiated into osteoclasts and treated with exogenous UD. UD supplementation significantly increased intracellular UD levels (**Figure 2A**; **Supplementary Figure 2A**). CCK-8 assays showed that 0–4 mM UD had no effect on cell viability, indicating good cytocompatibility within this concentration range (**Figure 2B**; **Supplementary Figure 2B**).

Functional assays revealed that UD effectively suppressed RANKL-induced osteoclast formation. TRAP staining showed fewer multinucleated osteoclasts at higher UD concentrations (**Figures 2C, D**; **Supplementary Figures 2C–E**). The F-actin ring structure was disrupted, and bone resorption assays similarly showed a reduction in the resorption area (**Figures 2C, E, F**). At the molecular level, mRNA and protein expression of CTSK, NFATc1, and Acp5 were inhibited upon UD treatment, and the nuclear localization of NFATc1 was significantly reduced (**Figures 2G–I**). Collectively, these results indicate that exogenous UD inhibits RANKL-induced osteoclast differentiation and attenuates bone resorptive activity.

### 3.3 UD alleviated bone loss and suppressed osteoclast activation *in vivo*

**Figure 3:**
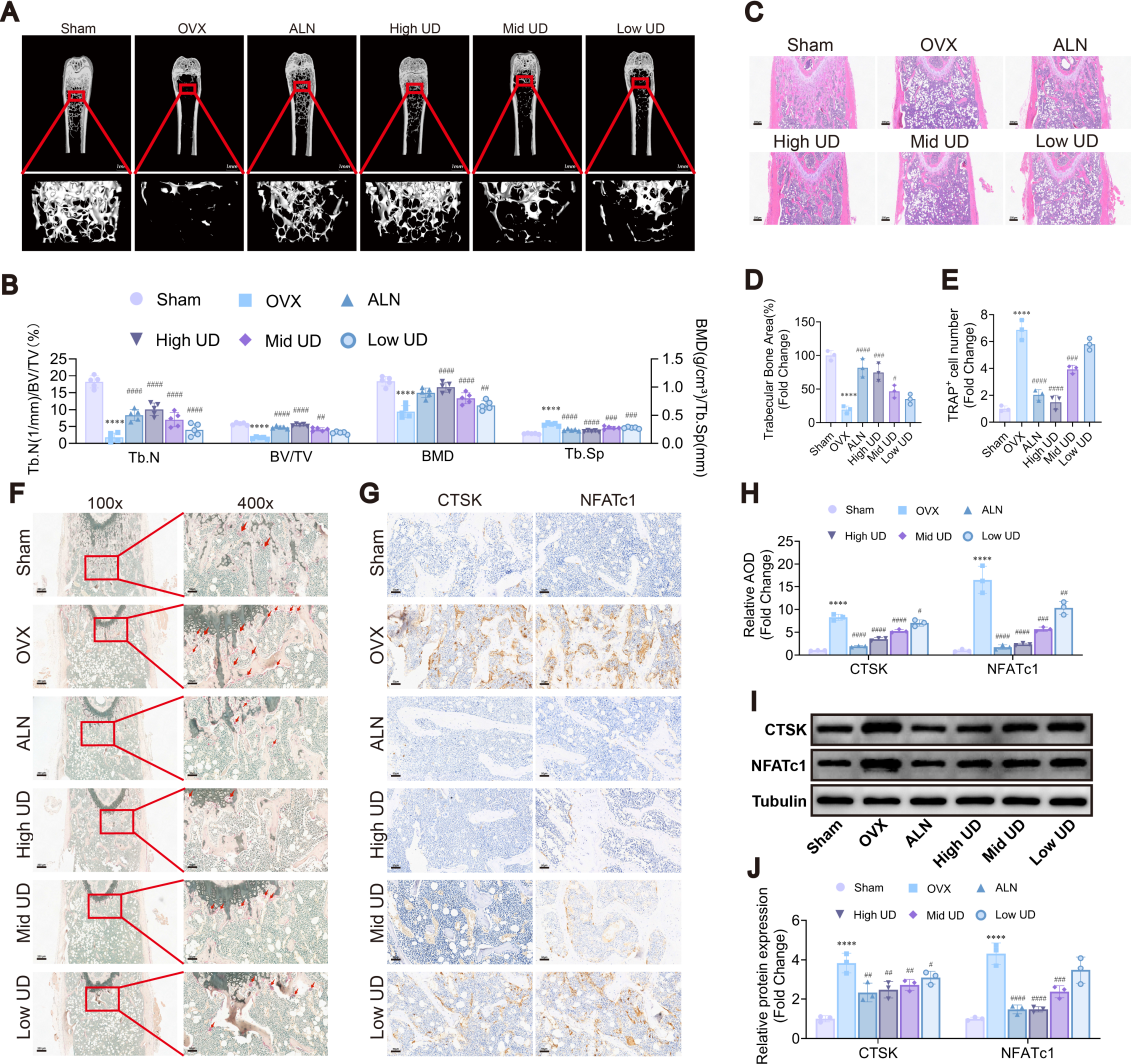
Uridine alleviated OVX-induced bone loss *in vivo*. **(A)** Representative micro-CT images of femurs, Scale bar = 1 mm; **(B)** Quantitative analysis of BMD, BV/TV, Tb.N, and Tb.Sp; **(C, D)** H&E staining and quantitative analysis of trabecular bone area, Scale bar = 500 μm; **(E, F)** TRAP staining and quantification of TRAP^+^ osteoclasts, Scale bar = 500 μm (low magnification) and 100 μm (high magnification); **(G, H)** Immunohistochemical staining and AOD of CTSK and NFATc1, Scale bar = 100 μm; **(I, J)** Western blot and densitometric analysis of CTSK and NFATc1. Data represent mean ± SD (n = 5). *p* < 0.05, *p < 0.01, **p < 0.001 vs. Sham; #*p* < 0.05, ##*p* < 0.01, ###*p* < 0.001 vs. OVX.

In this study, an OVX-induced osteoporosis mouse model was used to investigate the *in vivo* effects of UD. Micro-CT 3D reconstruction revealed that the OVX group exhibited sparse and disorganized trabecular architecture, whereas treatment with ALN or UD restored trabecular integrity and density (**Figure 3A**). Quantitative analysis showed significant reductions in trabecular number (Tb.N), bone volume fraction (BV/TV), and bone mineral density (BMD), along with an increase in trabecular separation (Tb.Sp) in the OVX group compared to the sham group. UD treatment effectively reversed these changes, with the high-dose group showing improvements comparable to ALN (**Figure 3B**).

Histological examination supported the micro-CT findings, as OVX mice displayed reduced and disorganized trabecular bone, while UD treatment resulted in denser and more continuous structures. Quantification confirmed a significant increase in trabecular bone area after UD treatment (**Figures 3C, D**). TRAP staining revealed an obvious increase in osteoclast number in OVX mice, which was significantly reduced by UD treatment (**Figures 3E, F**). Immunohistochemistry demonstrated elevated expression of CTSK and NFATc1 in OVX bone tissue, both of which were notably decreased after UD administration (**Figures 3G, H**). Western blot analysis further confirmed that UD downregulated the protein levels of CTSK and NFATc1 in a dose-dependent manner, with the highest dose showing the strongest inhibitory effect (**Figures 3I, J**). Collectively, these results indicate that exogenous UD effectively mitigates OVX-induced bone loss by suppressing excessive osteoclast activation and reducing the expression of key osteoclast-related proteins *in vivo*.

### 3.4 UD inhibited PI3K/Akt signaling and promoted FoxO activation

**Figure 4:**
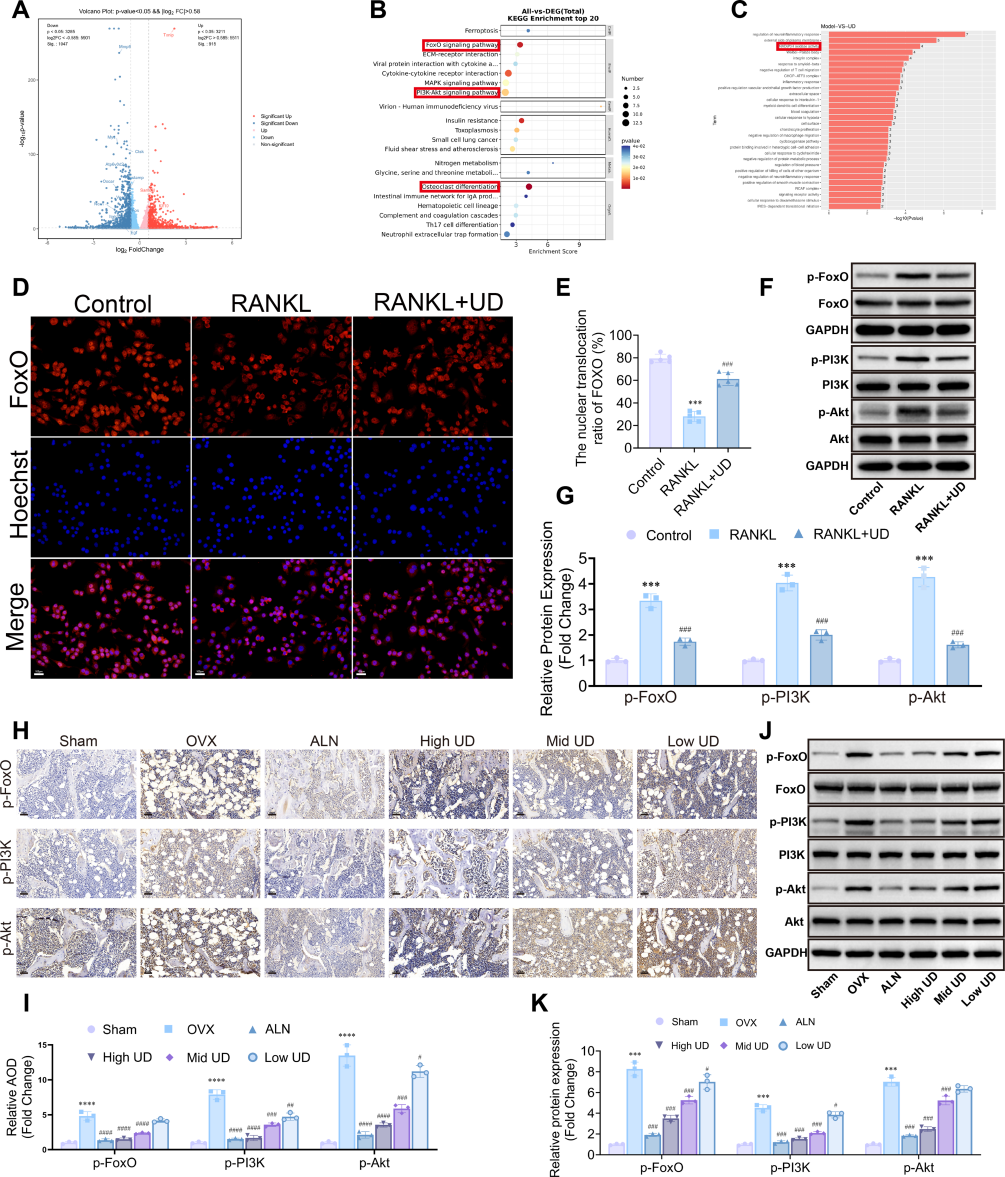
Uridine inhibited PI3K/Akt and activated FoxO. **(A)** Volcano plot of DEGs between the model and uridine groups; **(B, C)** KEGG and GO enrichment analyses; **(D, E)** Immunofluorescence staining and quantification of FoxO nuclear translocation, Scale bar = 100 μm; **(F, G)** Western blot and densitometric analysis of total and phosphorylated FoxO, PI3K, and Akt *in vitro*; **(H, I)** Immunohistochemical staining and AOD of p-FoxO, p-PI3K, and p-Akt in bone tissue; **(J, K)** Western blot and densitometric analysis of p-FoxO, p-PI3K, and p-Akt in bone tissue, Scale bar = 100 μm. Data represent mean ± SD (n = 3). *p < 0.05, **p < 0.01, ***p < 0.001 vs. Control (Sham); #*p* < 0.05, ##*p* < 0.01, ###*p* < 0.001 vs. RANKL (OVX).

To explore how UD inhibits osteoclast differentiation, transcriptomic analysis was performed on control, RANKL-induced, and UD-treated groups. PCA revealed a clear separation in the transcriptional profiles among the three groups (**Supplementary Figure 3A**). Volcano plots and KEGG analysis showed that DEGs were primarily enriched in osteoclast differentiation, PI3K/Akt, and FoxO signaling pathways (**Figures 4A, B**). GO analysis further indicated enrichment in processes related to oxidative stress (**Figure 4C**). GSEA confirmed that UD significantly suppressed the enrichment of gene sets associated with osteoclast differentiation and the PI3K/Akt signaling pathway (**Supplementary Figures 3B, C**).

At the cellular level, RANKL treatment reduced the nuclear localization of FoxO (36), whereas UD restored its nuclear translocation (**Figures 4D, E**). Western blot analysis revealed that RANKL significantly upregulated the levels of p-PI3K, p-Akt, and p-FoxO, while UD treatment markedly inhibited the phosphorylation of these proteins (**Figures 4F, G**). These findings suggest that UD blocks the activation of PI3K/Akt signaling and prevents FoxO phosphorylation.

*In vivo*, levels of p-FoxO, p-PI3K, and p-Akt were elevated in OVX bone tissue. UD treatment downregulated these levels in a dose-dependent manner (**Figures 4H, I**). Immunohistochemistry results confirmed these findings, showing reduced p-PI3K/PI3K, p-Akt/Akt, and p-FoxO/FoxO ratios after UD treatment (**Figures 4J, K**).

Collectively, these results indicate that UD inhibits osteoclast differentiation both *in vitro* and *in vivo* by suppressing PI3K/Akt signaling activation and preventing FoxO phosphorylation.

### 3.5 UD inhibited osteoclast differentiation by eliminating ROS

**Figure 5:**
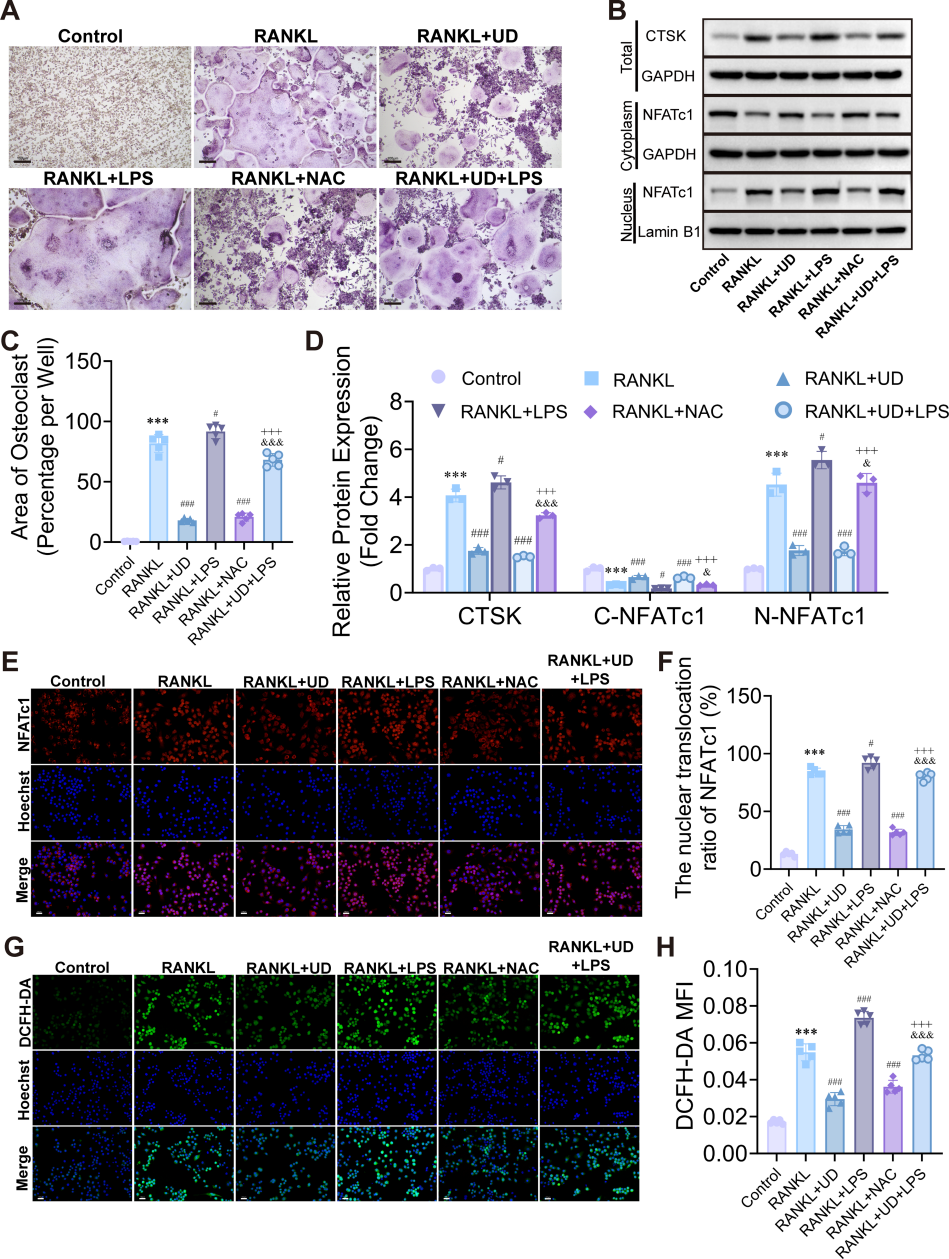
UD inhibited osteoclast differentiation by attenuating ROS accumulation. **(A, C)** TRAP staining and quantification of osteoclast area under different treatments, Scale bar = 100 μm; **(B, D)** Western blot and quantification of CTSK and cytoplasmic/nuclear NFATc1; **(E, F)** Immunofluorescence staining and quantification of NFATc1 nuclear translocation, Scale bar = 100 μm; **(G, H)** DCFH-DA fluorescence staining and quantification of intracellular ROS, Scale bar = 100 μm. Data represent mean ± SD (n = 3). *p < 0.05, **p < 0.01, ***p < 0.001 vs. Control; #*p* < 0.05, ##*p* < 0.01, ###*p* < 0.001 vs. RANKL; &*p* < 0.05, &&*p* < 0.01, &&&*p* < 0.001 vs. RANKL + UD; +*p* < 0.05, ++*p* < 0.01, +++*p* < 0.001 vs. RANKL + LPS.

Transcriptomic GO enrichment analysis suggested that oxidative stress may be involved in the inhibitory effect of UD on osteoclast differentiation. ROS has been recognized as an essential mediator in osteoclast differentiation. To test this hypothesis, we explored the functional involvement of ROS. TRAP staining revealed that UD significantly suppressed osteoclast formation, an effect that was partially reversed by the ROS agonist LPS. In contrast, treatment with the ROS scavenger NAC produced similar results to UD, further supporting the critical role of oxidative stress in osteoclast differentiation (**Figures 5A, C**).

At the molecular level, Western blot (**Figures 5B, D**) and immunofluorescence (**Figures 5E, G**) analyses confirmed that UD downregulated the expression of CTSK and inhibited the nuclear translocation of NFATc1. LPS partially restored these effects, whereas NAC maintained an inhibitory pattern similar to that of UD. Additionally, ROS fluorescence detection demonstrated that LPS reversed the ROS levels reduced by UD, while NAC exhibited a similar antioxidative effect (**Figures 5F, H**). Together, these findings indicate that changes in ROS levels are closely associated with NFATc1 activation and CTSK expression, suggesting that ROS serves as a key mediator in the UD-induced inhibition of osteoclast differentiation.

### 3.6 UD inhibited osteoclast differentiation via the PI3K/Akt–FoxO–ROS pathway

**Figure 6:**
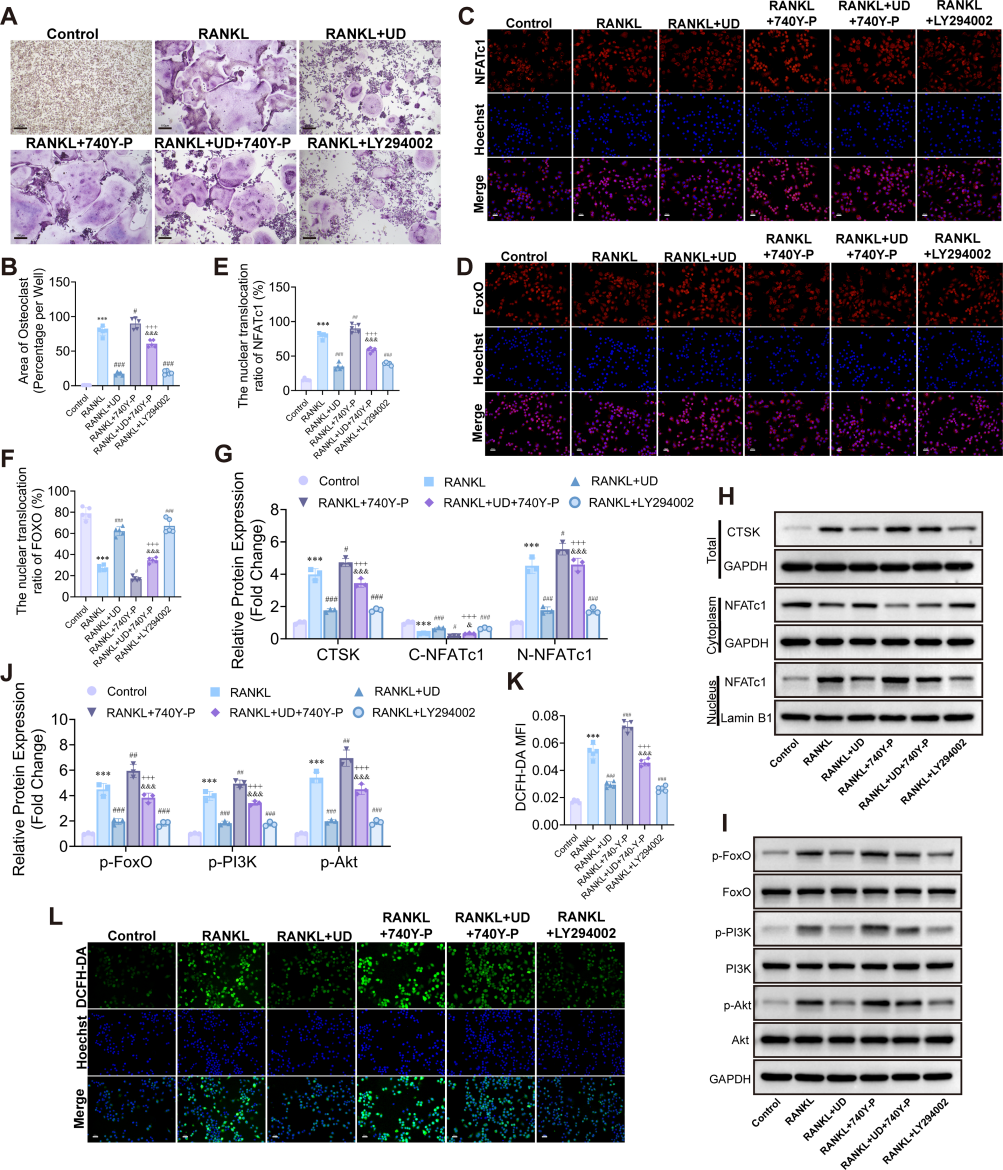
UD suppressed osteoclast differentiation by modulating PI3K/Akt–FoxO signaling. **(A, B)** TRAP staining and quantification of osteoclast area under different treatments; **(C–F)** Immunofluorescence staining and quantification of NFATc1 and FoxO nuclear translocation, Scale bar = 100 μm; **(G, H)** Western blot and quantification of CTSK and cytoplasmic/nuclear NFATc1; **(I, J)** Western blot and quantification of phosphorylated PI3K, Akt, and FoxO; **(K, L)** DCFH-DA fluorescence staining and quantification of intracellular ROS, Scale bar = 100 μm. Data represent mean ± SD (n = 3). *p < 0.05, **p < 0.01, ***p < 0.001 vs. Control; #*p* < 0.05, ##*p* < 0.01, ###*p* < 0.001 vs. RANKL; &*p* < 0.05, &&*p* < 0.01, &&&*p* < 0.001 vs. RANKL + UD; +*p* < 0.05, ++*p* < 0.01, +++*p* < 0.001 vs. RANKL + 740Y-P.

Given that FoxO activation plays a critical role in ROS elimination, we investigated whether the PI3K/Akt–FoxO pathway mediates the inhibitory effects of UD on osteoclast differentiation using the PI3K agonist 740Y-P and inhibitor LY294002. TRAP staining revealed that UD significantly reduced RANKL-induced osteoclast formation, a suppression partially reversed by PI3K activation. In contrast, PI3K inhibition mimicked the effects of UD treatment (**Figures 6A, D**). Immunofluorescence analysis demonstrated that UD promoted the nuclear translocation of FoxO and inhibited the nuclear localization of NFATc1. These effects were attenuated upon PI3K activation, while PI3K inhibition preserved UD’s regulatory influence (**Figures 6B, C, E, F**). Western blot analysis confirmed that UD downregulated the expression of CTSK and NFATc1, and reduced the levels of p-PI3K, p-Akt, and p-FoxO. Activation of the PI3K pathway with 740Y-P partially reversed these changes, while LY294002 reproduced the effects of UD (**Figures 6G–J**).

Given the close association between this signaling pathway and oxidative stress, as indicated by transcriptomic data, we further examined intracellular ROS levels. RANKL induced significant ROS accumulation, which was markedly reduced by UD treatment. However, this antioxidative effect was attenuated with PI3K activation, whereas pathway inhibition maintained the suppressive trend (**Figures 6K, L**). Collectively, these results demonstrate that UD inhibits osteoclast differentiation by suppressing PI3K/Akt signaling, promoting FoxO nuclear function, and alleviating oxidative stress. This coordinated regulation across signaling, transcriptional, and functional levels is crucial for its inhibitory effects on osteoclast differentiation.

## 4 Discussion

Osteoporosis is a metabolic skeletal disorder characterized by an imbalance between bone formation and resorption, with excessive osteoclast activity serving as a major driver of pathogenesis (37). Recent evidence indicates that osteoclast differentiation is regulated not only by classical signaling pathways but also by cellular metabolic dynamics (37–39). In this study, we used multi-omics analyses to explore the metabolic and transcriptional changes associated with RANKL-induced osteoclast differentiation (40). Our findings revealed significant metabolic reprogramming, with pyrimidine metabolism emerging as a key pathway consistently enriched in both datasets. This suggests that dysregulated nucleotide metabolism plays a crucial role in osteoclast differentiation and the development of osteoporosis. These insights highlight the importance of metabolic mechanisms in osteoclast differentiation and open new avenues for therapeutic exploration in osteoporosis.

Previous studies have highlighted the crucial role of metabolic reprogramming in osteoclast differentiation and bone homeostasis, particularly through pathways related to energy and lipid metabolism (23, 37). In this study, RANKL-induced osteoclast differentiation was similarly associated with substantial metabolic alterations. Interestingly, in addition to conventional energy metabolism, pyrimidine metabolism also exhibited significant dysregulation, within which uridine exhibited the most pronounced decrease among pyrimidine-related metabolites. As a metabolically active nucleoside directly involved in nucleotide synthesis, uridine was therefore prioritized for further investigation (41). In this context, the dosing of uridine was determined based on previous studies as well as our prior experimental findings (35, 42), and short-term safety evaluation revealed no apparent adverse effects (**Supplementary Figure 4**). Our findings revealed that osteoclast differentiation led to a marked decrease in UD levels, whereas exogenous UD supplementation restored metabolic balance, suppressed osteoclast formation, and alleviated bone loss. This suggests that UD deficiency may be a key factor driving the disruption of pyrimidine metabolism during osteoclast differentiation. Recent research has further confirmed that UD not only serves as an important intermediate in pyrimidine metabolism but is also involved in various physiological processes, including hepatic lipid metabolism, neuroprotection, and immune homeostasis (43–45). From the perspective of bone metabolism, our study highlights the potential role of UD in maintaining osteoclast metabolic homeostasis and regulating bone remodeling. These findings suggest that restoring intracellular UD metabolism could offer protective effects against the development and progression of osteoporosis by correcting abnormal osteoclast differentiation.

In bone metabolic homeostasis, ROS are critical molecules that connect metabolic status to osteoclastic activity (46). At physiological levels, ROS promote RANKL signaling and osteoclast differentiation. However, sustained oxidative stress exacerbates osteoclastogenic signals, disrupting the balance between bone resorption and formation (47). Previous studies have indicated a compromised antioxidant system as a hallmark of postmenopausal osteoporosis, and enhancing antioxidant capacity has been shown to significantly improve bone mass (48, 49). Based on these insights, our study demonstrated that UD inhibited osteoclast differentiation by reducing intracellular ROS levels, suggesting that its anti-resorptive effect is closely associated with redox homeostasis. UD, as an intermediate in pyrimidine metabolism, may exert its antioxidant effects not through direct free radical scavenging, but rather by remodeling cellular metabolic processes (50, 51). Pyrimidine metabolism plays a crucial role in regulating NAD(P)H balance and mitochondrial function, both of which directly influence ROS production and clearance. Therefore, UD may suppress persistent oxidative stress by restoring cellular energy balance and maintaining redox homeostasis at the metabolic level.

Within the complex interplay between metabolism and signaling networks, the PI3K/Akt–FoxO pathway plays a pivotal role in linking cellular energy status to osteoclast activity (36, 52, 53). This study revealed that UD did not simply act as a metabolic supplement, but actively remodeled the connection between cellular signaling and redox regulation, thereby suppressing osteoclast activity. By suppressing PI3K/Akt signaling and activating FoxO, UD shifts signal transduction and bolsters cellular antioxidant defenses, leading to a reduction in ROS accumulation. In osteoclasts, FoxO-mediated control of redox homeostasis represents a key checkpoint through which upstream signaling activity translates into ROS-dependent regulation of osteoclast differentiation. Our findings highlight how metabolic remodeling can feedback-regulate signaling pathways through FoxO-mediated antioxidant functions, establishing a synergistic balance between metabolism, signaling, and ROS. Thus, osteoclast differentiation is not driven solely by signaling pathways, but by a dynamic equilibrium influenced by both metabolic regulation and oxidative stress. Moreover, the modulation of the PI3K/Akt–FoxO–ROS axis through pyrimidine metabolism offers a novel theoretical framework for targeting osteoclast activation by metabolic intervention.

From a mechanistic perspective, the coordinated regulation of metabolism, redox homeostasis, and signaling pathways identified in this study highlights a tightly interconnected network underlying osteoclast differentiation, with the PI3K/Akt–FoxO–ROS axis serving as a central integrative node (54). From a mechanistic perspective, the coordinated regulation of metabolism, redox homeostasis, and signaling pathways identified in this study highlights a tightly interconnected network underlying osteoclast differentiation, with the PI3K/Akt–FoxO–ROS axis serving as a central integrative node (55–57). These findings suggest that FoxO functions as a key hub linking metabolic status, oxidative stress, and osteoclastogenic signaling. Although the present study focuses on the PI3K/Akt–FoxO axis, uridine-induced metabolic remodeling may intersect with these additional regulatory layers, further supporting a role for metabolic regulation in osteoclast differentiation.

**Figure 7:**
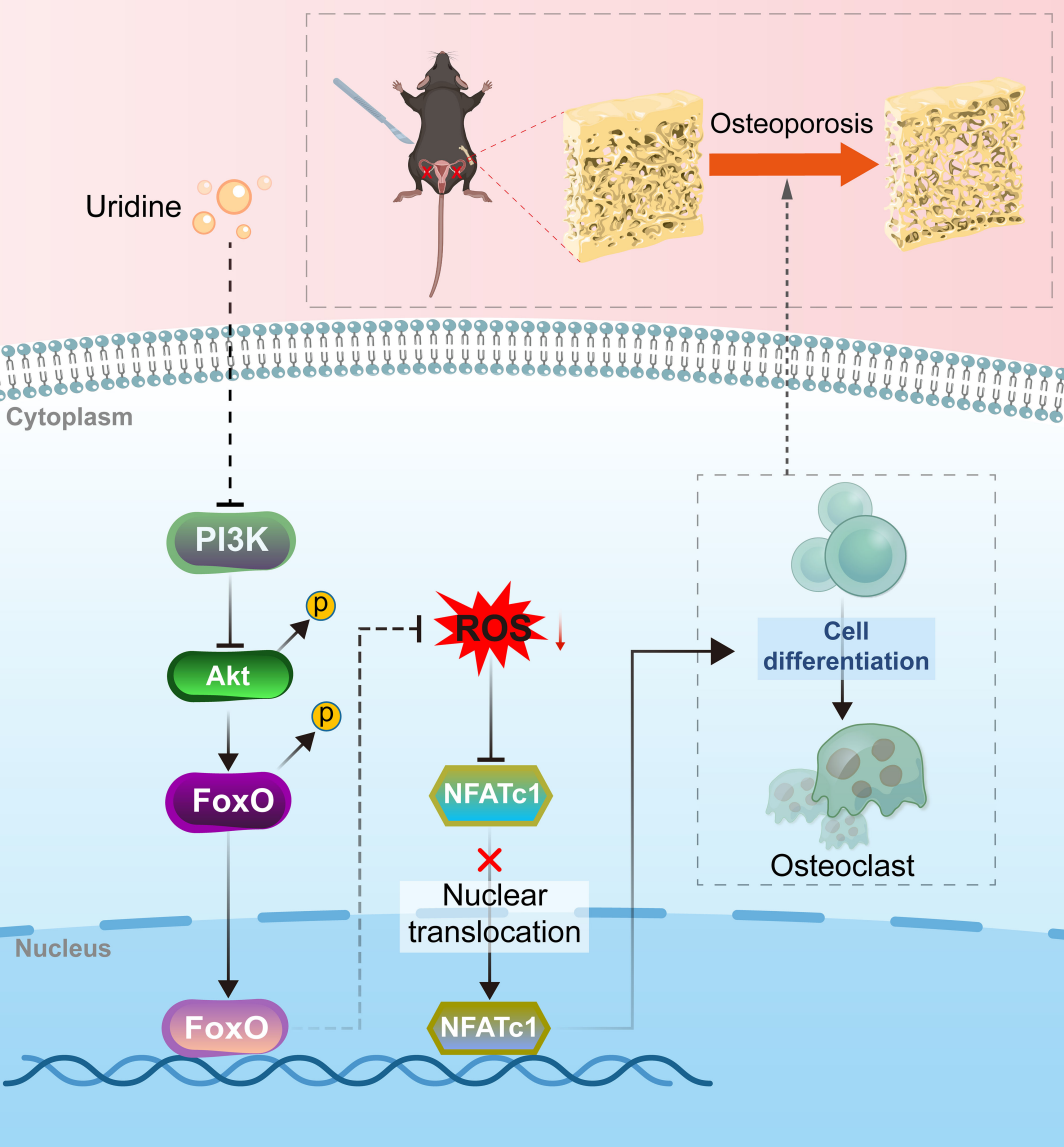
Proposed mechanism of uridine in inhibiting osteoclast differentiation. Uridine suppresses osteoclast differentiation by inhibiting PI3K/Akt activation, enhancing FoxO activity, reducing ROS accumulation, and blocking NFATc1 nuclear translocation, ultimately preventing osteoclast formation and bone resorption.

UD inhibits osteoclast differentiation via the PI3K/Akt–FoxO–ROS pathway, providing a new perspective on the role of metabolic regulation in bone resorption, as schematically illustrated in **Figure 7**. Future studies could expand on these findings by investigating the long-term effects of UD in different types of osteoporosis models, or validating its sustained impact over extended periods. Additionally, exploring the synergistic role of UD and its related metabolites within the pyrimidine metabolic network could provide deeper insights. From a broader viewpoint, UD not only participates in energy and substrate metabolism but also maintains cellular redox homeostasis by regulating the PI3K/Akt–FoxO axis. This regulation suppresses excessive ROS accumulation and NFATc1 activation, ultimately inhibiting osteoclast differentiation. These findings suggest that the relationship between metabolism and signaling is bidirectional, forming a dynamically interactive regulatory network. This provides a novel research direction for investigating bone resorption mechanisms from a metabolic perspective.

## 5 Conclusion

In summary, this study identified UD as a critical link between pyrimidine metabolism and osteoclast differentiation. By suppressing PI3K/Akt phosphorylation, promoting FoxO nuclear localization, and reducing ROS accumulation, UD establishes a coupling mechanism between metabolism and signaling that restrains NFATc1-driven osteoclast activation. These findings unveil a novel regulatory axis governing bone resorption and provide a metabolic rationale for developing pyrimidine-based interventions in osteoporosis treatment.

## Data Availability

The datasets presented in this study can be found in online repositories. The names of the repository/repositories and accession number(s) can be found below: GSE311708 and GSE311709 (GEO) and MTBLS13394 (Metabolights).

## References

[1] Yang J Zeng Y Yu W . Criteria for osteoporosis diagnosis: a systematic review and meta-analysis of osteoporosis diagnostic studies with DXA and QCT. EClinicalMedicine. (2025) 83:103244. doi: 10.1016/j.eclinm.2025.103244. PMID: 40630613 PMC12235398

[2] Ensrud KE Crandall CJ . Osteoporosis. Ann Intern Med. (2024) 177:ITC1–ITC16. doi: 10.1001/jama.2020.2923. PMID: 38190715

[3] Collaborators GBDLBMD . The global, regional, and national burden attributable to low bone mineral density, 1990-2020: an analysis of a modifiable risk factor from the Global Burden of Disease Study 2021. Lancet Rheumatol. (2025) 7(12):e873–e894. doi: 10.2139/ssrn.4771393. PMID: 40972625 PMC12623303

[4] Abbasi FM Porwal K Pal S Sharma S Sadhukhan S Rajput S . Discovery of an orally active PDE1 inhibitor for disease-modifying treatment of postmenopausal osteoporosis via dual anabolic-antiresorptive mechanisms. J Med Chem. (2025) 68:20536–60. doi: 10.1021/acs.jmedchem.5c01736. PMID: 41002212

[5] Wu Q Dai J . Racial/ethnic differences in bone mineral density for osteoporosis. Curr Osteoporos Rep. (2023) 21:670–84. doi: 10.1007/s11914-023-00838-y. PMID: 38019343

[6] Xie Q Du X Liang J Shen Y Ling Y Huang Z . FABP4 inhibition suppresses bone resorption and protects against postmenopausal osteoporosis in ovariectomized mice. Nat Commun. (2025) 16:4437. doi: 10.1038/s41467-025-59719-w. PMID: 40360512 PMC12075751

[7] Girdler SJ Lindsey MH Sebastian AS Nassr A . Osteoporosis evaluation and management in spine surgery. J Am Acad Orthop Surg. (2024) 32:e909–e18. doi: 10.5435/jaaos-d-24-00311. PMID: 39083525

[8] Wang H Luo Y Wang H Li F Yu F Ye L . Mechanistic advances in osteoporosis and anti-osteoporosis therapies. MedComm (2020). (2023) 4:e244. doi: 10.1002/mco2.244. PMID: 37188325 PMC10175743

[9] Reid IR Billington EO . Drug therapy for osteoporosis in older adults. Lancet. (2022) 399:1080–92. doi: 10.1016/s0140-6736(21)02646-5. PMID: 35279261

[10] Chandran M Akesson KE Javaid MK Harvey N Blank RD Brandi ML . Impact of osteoporosis and osteoporosis medications on fracture healing: a narrative review. Osteoporos Int. (2024) 35:1337–58. doi: 10.1007/s00198-024-07059-8. PMID: 38587674 PMC11282157

[11] Shi K Wang Y Wang S Guan Z Li M Zhao Y . Okanin attenuates ovariectomy-induced bone loss in mice model through inhibition of IKKβ-mediated NF-κB p65 phosphorylation. Phytomedicine. (2025) 148:157401. doi: 10.1016/j.phymed.2025.157401. PMID: 41109043

[12] Zhang H Zhao X Wang Z Miao J Hu X Cui P . Crebanine protects against ovariectomy-induced bone loss by targeting Sirt1 to interfere with NF-κB acetylation and ROS activity. J Pharm Anal. (2025) 16(3). doi: 10.1016/j.jpha.2025.101426. PMID: 41953925 PMC13054427

[13] Zhou H Chen P Zhao C Zou S Wu H Huang C . Fraxin inhibits ovariectomized-induced bone loss and osteoclastogenesis by suppressing ROS activity. Int Immunopharmacol. (2025) 147:113871. doi: 10.1016/j.intimp.2024.113871. PMID: 39798467

[14] Cui Z Gu G Chen F Li J Du X Chen S . Targeting Irgm1 to combat osteoporosis: suppressing ROS and restoring bone remodeling. Cell Death Dis. (2025) 16:651. doi: 10.1038/s41419-025-07965-7. PMID: 40866352 PMC12391319

[15] Liang H Wu Q Hua T Chen Y Luo J Miao J . Sofalcone inhibits osteoclastogenesis through Keap1/Nrf2 signaling activation and mitigates ovariectomy-induced bone loss. Int Immunopharmacol. (2025) 166:115589. doi: 10.1016/j.intimp.2025.115589. PMID: 41005145

[16] Qiu H Cai C Zhang Y Yang S Hu X Chu T . Salvianolic acid A mitigates osteoporotic bone loss by repressing reactive oxygen species via the Nrf2-HO-1 pathway. Phytother Res. (2025) 39(11). doi: 10.1002/ptr.8503. PMID: 40765418 PMC12605832

[17] Yuan P Feng Z Yang H Xue H Xie H Dai Z . Visomitin attenuates pathological bone loss by reprogramming osteoclast metabolism via the STAT3/LDHB axis. Res (Wash D C). (2025) 8:784. doi: 10.34133/research.0784. PMID: 40698330 PMC12280330

[18] Mendelsohn DH Walter N Cheung WH Wong RMY Schonmehl R Winter L . Targeting mitochondria in bone and cartilage diseases: a narrative review. Redox Biol. (2025) 83:103667. doi: 10.1016/j.redox.2025.103667. PMID: 40354767 PMC12136924

[19] Rodriguez-Colman MJ Dansen TB Burgering BMT . FOXO transcription factors as mediators of stress adaptation. Nat Rev Mol Cell Biol. (2024) 25:46–64. doi: 10.1038/s41580-023-00649-0. PMID: 37710009

[20] Huang H van Sligtenhorst M Smits AMM Gulersonmez C Stigter E Dansen TB . Activation of a FOXO3-induced cell cycle arrest regulates ferroptosis. Cell Death Discov. (2025) 11:465. doi: 10.1038/s41420-025-02760-x. PMID: 41102177 PMC12533257

[21] Song J Li Z Zhou L Chen X Sew WQG Herranz H . FOXO-regulated OSER1 reduces oxidative stress and extends lifespan in multiple species. Nat Commun. (2024) 15:7144. doi: 10.1038/s41467-024-51542-z. PMID: 39164296 PMC11336091

[22] Wang C Wu Q Zhuang L Chen Y Zhang Q Wu Y . Immunometabolism of macrophages in the bone microenvironment: a new perspective for bone healing therapy. J Adv Res. (2025) 82:485–506. doi: 10.1016/j.jare.2025.07.046. PMID: 40744273 PMC13001043

[23] He T Qin L Chen S Huo S Li J Zhang F . Bone-derived factors mediate crosstalk between skeletal and extra-skeletal organs. Bone Res. (2025) 13:49. doi: 10.1038/s41413-025-00424-1. PMID: 40307216 PMC12044029

[24] Bohacova K Nahacka Z Dudova J Kovarova J Rohlena J Rennerova M . Role of ANT2 in mitochondrial function and cancer cell survival: a target for therapeutic intervention. Cell Death Discov. (2025) 11:225. doi: 10.1038/s41420-025-02510-z. PMID: 40335504 PMC12059193

[25] Chen CY Chen CL Ng YS Lee DY Lin SS Huang CK . Glucose- and glutamine-driven de novo nucleotide synthesis facilitates WSSV replication in shrimp. Cell Commun Signal. (2025) 23:191. doi: 10.1186/s12964-025-02186-z. PMID: 40264189 PMC12012963

[26] Mironova GD Mosentsov AA Mironov VV Medvedeva VP Khunderyakova NV Pavlik LL . The protective effect of uridine in a rotenone-induced model of Parkinson’s disease: the role of the mitochondrial ATP-dependent potassium channel. Int J Mol Sci. (2024) 25:13. doi: 10.3390/ijms25137441. PMID: 39000550 PMC11242281

[27] Lin Y Wei Y Wei Y Yu H Zhang W Li C . Dexmedetomidine alleviates oxidative stress and mitochondrial dysfunction in diabetic peripheral neuropathy via the microRNA-34a/SIRT2/S1PR1 axis. Int Immunopharmacol. (2023) 117:109910. doi: 10.1016/j.intimp.2023.109910. PMID: 37012886

[28] Yang Y Ye Y Deng Y Gao L . Uridine and its role in metabolic diseases, tumors, and neurodegenerative diseases. Front Physiol. (2024) 15:1360891. doi: 10.3389/fphys.2024.1360891. PMID: 38487261 PMC10937367

[29] Huang M Xing F Hu Y Sun F Zhang C Xv Z . Causal inference study of plasma proteins and blood metabolites mediating the effect of obesity-related indicators on osteoporosis. Front Endocrinol (Lausanne). (2025) 16:1435295. doi: 10.3389/fendo.2025.1435295. PMID: 40041284 PMC11876022

[30] Hou JL Yang WY Zhang Q Feng H Wang XB Li H . Integration of metabolomics and transcriptomics to reveal the metabolic characteristics of exercise-improved bone mass. Nutrients. (2023) 15:7. doi: 10.3390/nu15071694. PMID: 37049535 PMC10097349

[31] Zhao H Li X Zhang D Chen H Chao Y Wu K . Integrative bone metabolomics-lipidomics strategy for pathological mechanism of postmenopausal osteoporosis mouse model. Sci Rep. (2018) 8:16456. doi: 10.1038/s41598-018-34574-6. PMID: 30405156 PMC6220250

[32] Zhao Y Ning J Teng H Deng Y Sheldon M Shi L . Long noncoding RNA Malat1 protects against osteoporosis and bone metastasis. Nat Commun. (2024) 15:2384. doi: 10.1038/s41467-024-46602-3. PMID: 38493144 PMC10944492

[33] Son HS Lee J Lee HI Kim N Jo YJ Lee GR . Benzydamine inhibits osteoclast differentiation and bone resorption via down-regulation of interleukin-1 beta expression. Acta Pharm Sin B. (2020) 10:462–74. doi: 10.1016/j.apsb.2019.11.004. PMID: 32140392 PMC7049613

[34] Yu M Pal S Paterson CW Li JY Tyagi AM Adams J . Ovariectomy induces bone loss via microbial-dependent trafficking of intestinal TNF+ T cells and Th17 cells. J Clin Invest. (2021) 131:4. doi: 10.1172/jci143137. PMID: 33586672 PMC7880410

[35] Zhou X Xue Q Wu C Li X Wang Y Dai Y . A novel combination therapy with uridine and praziquantel effectively alleviates schistosomiasis-induced hepatic fibrosis through promoting adipogenic differentiation. PloS Pathog. (2025) 21:e1013403. doi: 10.1371/journal.ppat.1013403. PMID: 40768528 PMC12349724

[36] Chai S Yang Y Wei L Cao Y Ma J Zheng X . Luteolin rescues postmenopausal osteoporosis elicited by OVX through alleviating osteoblast pyroptosis via activating PI3K-AKT signaling. Phytomedicine. (2024) 128:155516. doi: 10.1016/j.phymed.2024.155516. PMID: 38547625

[37] Stegen S Moermans K Stockmans I Thienpont B Carmeliet G . The serine synthesis pathway drives osteoclast differentiation through epigenetic regulation of NFATc1 expression. Nat Metab. (2024) 6:141–52. doi: 10.1038/s42255-023-00948-y. PMID: 38200114 PMC10822776

[38] Qiu H Jin H Miao J Li H Chen J Yang X . Heme metabolism mediates RANKL-induced osteoclastogenesis via mitochondrial oxidative phosphorylation. J Bone Miner Res. (2025) 40:639–55. doi: 10.1093/jbmr/zjaf040. PMID: 40073838 PMC12103724

[39] Ledesma-Colunga MG Passin V Lademann F Hofbauer LC Rauner M . Novel insights into osteoclast energy metabolism. Curr Osteoporos Rep. (2023) 21:660–9. doi: 10.1007/s11914-023-00825-3. PMID: 37816910 PMC10724336

[40] Huan C Li J Li Y Zhao S Yang Q Zhang Z . Spatially resolved multiomics: data analysis from monoomics to multiomics. BME Front. (2025) 6:84. doi: 10.34133/bmef.0084. PMID: 39810754 PMC11725630

[41] Choi K-M Berard BA Yoon J-H Kim D . Uridine as a hub in cancer metabolism and RNA biology. Exp Mol Med. (2025) 57:1651–62. doi: 10.1038/s12276-025-01402-7. PMID: 40804482 PMC12411634

[42] Goren B Cakir A Sevinc C Serter Kocoglu S Ocalan B Oy C . Uridine treatment protects against neonatal brain damage and long-term cognitive deficits caused by hyperoxia. Brain Res. (2017) 1676:57–68. doi: 10.1016/j.brainres.2017.09.010. PMID: 28919465

[43] Liu Y Xie C Zhai Z Deng ZY De Jonge HR Wu X . Uridine attenuates obesity, ameliorates hepatic lipid accumulation and modifies the gut microbiota composition in mice fed with a high-fat diet. Food Funct. (2021) 12:1829–40. doi: 10.1039/d0fo02533j. PMID: 33527946

[44] Corry KA White OR Shearlock AE Moralejo DH Law JB Snyder JM . Evaluating neuroprotective effects of uridine, erythropoietin, and therapeutic hypothermia in a ferret model of inflammation-sensitized hypoxic-ischemic encephalopathy. Int J Mol Sci. (2021) 22:18. doi: 10.3390/ijms22189841. PMID: 34576001 PMC8469346

[45] Whyte D Fisher SL McKenzie CGJ Sumpton D Dhayade S Dornier E . Uridine phosphorylase-1 supports metastasis by altering immune and extracellular matrix landscapes. EMBO Rep. (2025) 26:4248–82. doi: 10.1038/s44319-025-00520-7. PMID: 40702342 PMC12420820

[46] Gao Y Zhou R Lin X Liu Z Su Y Lian H . Sinensetin serves as an AMPK activator to inhibit RANKL-induced osteoclastogenesis via osteoclast cytoskeleton reorganization. J Transl Med. (2025) 23:805. doi: 10.1186/s12967-025-06708-8. PMID: 40682116 PMC12275404

[47] Lai Y Li H Zhou T Chen H Yang J Mo G . Epimedin B attenuates ovariectomy-induced bone loss by suppressing osteoclastogenesis through decreasing ROS production and targeting ESR1. Free Radic Biol Med. (2025) 240:347–63. doi: 10.1016/j.freeradbiomed.2025.08.033. PMID: 40834910

[48] Zhen C Wang S Yang J Zhang G Cai C Wang J . Moderate static magnetic field regulates iron metabolism and salvage bone loss caused by iron accumulation. J Orthopaedic Translation. (2025) 50:144–57. doi: 10.1016/j.jot.2024.10.012. PMID: 40171108 PMC11960543

[49] Yu C Cai L Zhuang L Wu Y Wu Q Liang H . Idebenone attenuates RANKL-induced osteoclastogenesis and improves bone mass in ovariectomized mice. Free Radic Biol Med. (2025) 238:206–19. doi: 10.1016/j.freeradbiomed.2025.06.043. PMID: 40578539

[50] Xiu C Zhang L Zhang C Zhang Y Luo X Zhang Z . Pharmacologically targeting fatty acid synthase-mediated de novo lipogenesis alleviates osteolytic bone loss by directly inhibiting osteoclastogenesis through suppression of STAT3 palmitoylation and ROS signaling. Metabolism. (2025) 167:156186. doi: 10.1016/j.metabol.2025.156186. PMID: 40081616

[51] Wang J Zhang Y Cao J Wang Y Anwar N Zhang Z . The role of autophagy in bone metabolism and clinical significance. Autophagy. (2023) 19:2409–27. doi: 10.1080/15548627.2023.2186112. PMID: 36858962 PMC10392742

[52] Zhao Q Li Y Zhang J Lei X Tang J Chen J . Bienzyme-engineered fibrous membranes: A mitochondrial-targeted strategy to reverse bone loss in osteoporotic models. Adv Fiber Mater. (2025) 7:1803–29. doi: 10.1007/s42765-025-00580-3. PMID: 41933263

[53] Zhou R Guo Q Xiao Y Guo Q Huang Y Li C . Endocrine role of bone in the regulation of energy metabolism. Bone Res. (2021) 9:25. doi: 10.1038/s41413-021-00142-4. PMID: 34016950 PMC8137703

[54] Wang G . Making “CASES” for AI in medicine. BME Front. (2024) 5:36. doi: 10.34133/bmef.0036. PMID: 38288398 PMC10823727

[55] Wang W Wang L Zhang B Shang S Zhao C Zhang W . 3D printing of personalized magnesium composite bone tissue engineering scaffold for bone and angiogenesis regeneration. Chem Eng J. (2024) 484:149444. doi: 10.1016/j.cej.2024.149444. PMID: 41936479

[56] Link W Ferreira BI . FOXO transcription factors: A brief overview. Methods Mol Biol (Clifton NJ). (2025) 2871:1–8. doi: 10.1007/978-1-0716-4217-7_1. PMID: 39565573

[57] Zhang W Li W Du J Yang C Yu L Yang P . Dnmt3a-mediated hypermethylation of FoxO3 promotes redox imbalance during osteoclastogenesis. Proc Natl Acad Sci USA. (2025) 122:e2418023122. doi: 10.1073/pnas.2418023122. PMID: 40106360 PMC11962505

