# Supplementary material for: Uridine inhibits ROS-mediated osteoclast differentiation and alleviates osteoporosis via modulation of PI3K/Akt–FoxO signaling
Source: Front Immunol. 2026 Apr 27;17:1767279. doi: 10.3389/fimmu.2026.1767279 (PMC13158086; doi:10.3389/fimmu.2026.1767279)
Supplement: Supplementary file 1 [file DataSheet1.docx]

Supplementary Material

# Supplementary Data

## Supplementary Data 1. Primer sequences used for quantitative real-time PCR (qRT-PCR)

| **Gene** | **Forward (5′→3′)** | **Reverse (5′→3′)** |
| --- | --- | --- |
| **CTSK** | CTCGGCGTTTAATTTGGGAGA | TCGAGAGGGAGGTATTCTGAGT |
| **NFATc1** | GGAGAGTCCGAGAATCGAGAT | TTGCAGCTAGGAAGTACGTCT |
| **Acp5** | TGTCATCTGTGAAAAGGTGGTC | ACTGGAGGACGCGTGTTATG |
| **GAPDH** | AGGGTCGGTGTGAACGGATTG | GGGGTCGTTGATGGCAACA |

## Supplementary Data 2. Abbreviation

| **Abbreviation** | **Full Name** | SDS-PAGE | Sodium dodecyl sulfate polyacrylamide gel electrophoresis |
| --- | --- | --- | --- |
| UD | Uridine | HRP | Horseradish peroxidase |
| RANKL | Receptor activator of nuclear factor-κB ligand | ECL | Enhanced chemiluminescence |
| NFATc1 | Nuclear factor of activated T cells c1 | PBS | Phosphate-buffered saline |
| CTSK | Cathepsin K | EDTA | Ethylenediaminetetraacetic acid |
| OVX | Ovariectomized | micro-CT | Micro-computed tomography |
| FoxO | Forkhead box O | PCA | Principal component analysis |
| PI3K | Phosphoinositide 3-kinase | OPLS-DA | Orthogonal partial least squares discriminant analysis |
| Akt | Protein kinase B | KEGG | Kyoto Encyclopedia of Genes and Genomes |
| ROS | Reactive oxygen species | GO | Gene Ontology |
| M-CSF | Macrophage colony-stimulating factor | GSEA | Gene set enrichment analysis |
| LPS | Lipopolysaccharide | DEGs | Differentially expressed genes |
| NAC | N-acetylcysteine | SD | Standard deviation |
| DMEM | Dulbecco’s modified Eagle medium | ALN | Alendronate |
| α-MEM | Alpha minimum essential medium | BMD | Bone mineral density |
| FBS | Fetal bovine serum | BV/TV | Bone volume fraction |
| CCK-8 | Cell Counting Kit-8 | Tb.N | Trabecular number |
| TRAP | Tartrate-resistant acid phosphatase | Tb.Th | Trabecular thickness |
| BMMs | Bone marrow–derived macrophages | Tb.Sp | Trabecular separation |
| ELISA | Enzyme-linked immunosorbent assay | AOD | Average optical density |

# Supplementary Figures and Tables

For more information on Supplementary Material and for details on the different file types accepted, please see [here](https://www.frontiersin.org/guidelines/author-guidelines#supplementary-material).

## Supplementary Figures


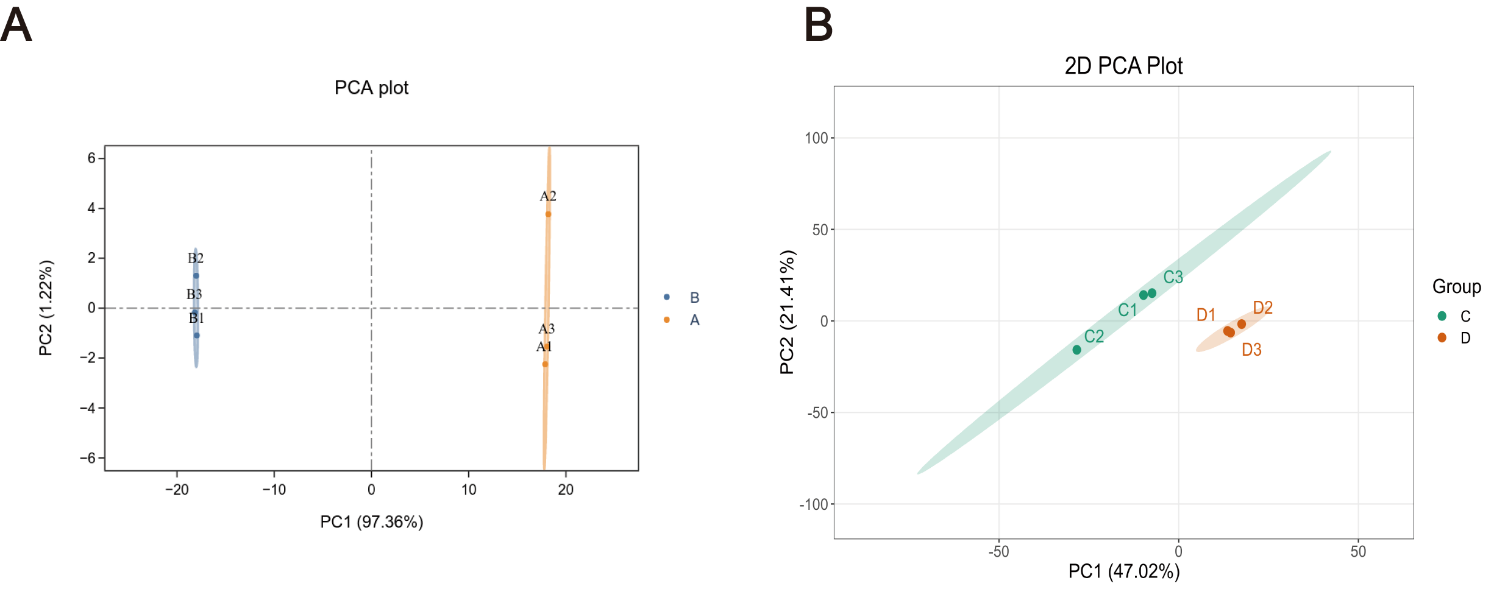


**Supplementary Figure S1.** **PCA analysis.**

(A, B) PCA plots of transcriptomic and metabolomic data.


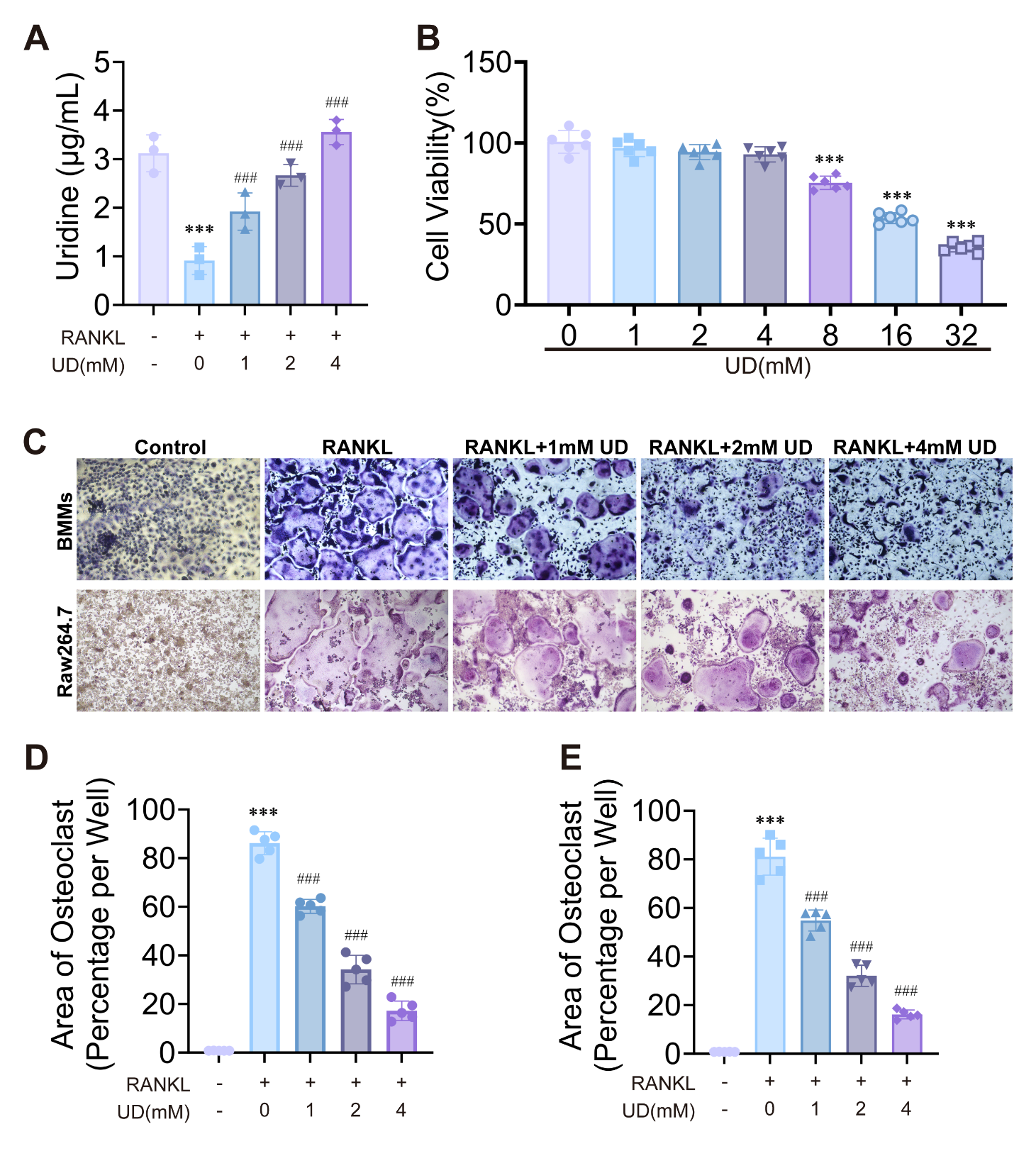


**Supplementary Figure S2.** **Effects of uridine on osteoclast differentiation and cell viability.**

1. Intracellular uridine levels in RAW264.7 cells after RANKL stimulation with or without UD treatment; (B) Cell viability of RAW264.7 cells treated with different UD concentrations for 48 h; (C) TRAP staining of BMMs and RAW264.7 cells showing osteoclast formation under different UD concentrations; (D, E) Quantification of TRAP⁺ osteoclast area in BMMs (D) and RAW264.7 cells (E). Data represent mean ± SD (n = 3). **p* < 0.05, ***p* < 0.01, ****p* < 0.001 vs. Control; #*p* < 0.05, ##*p* < 0.01, ###*p* < 0.001 vs. RANKL.


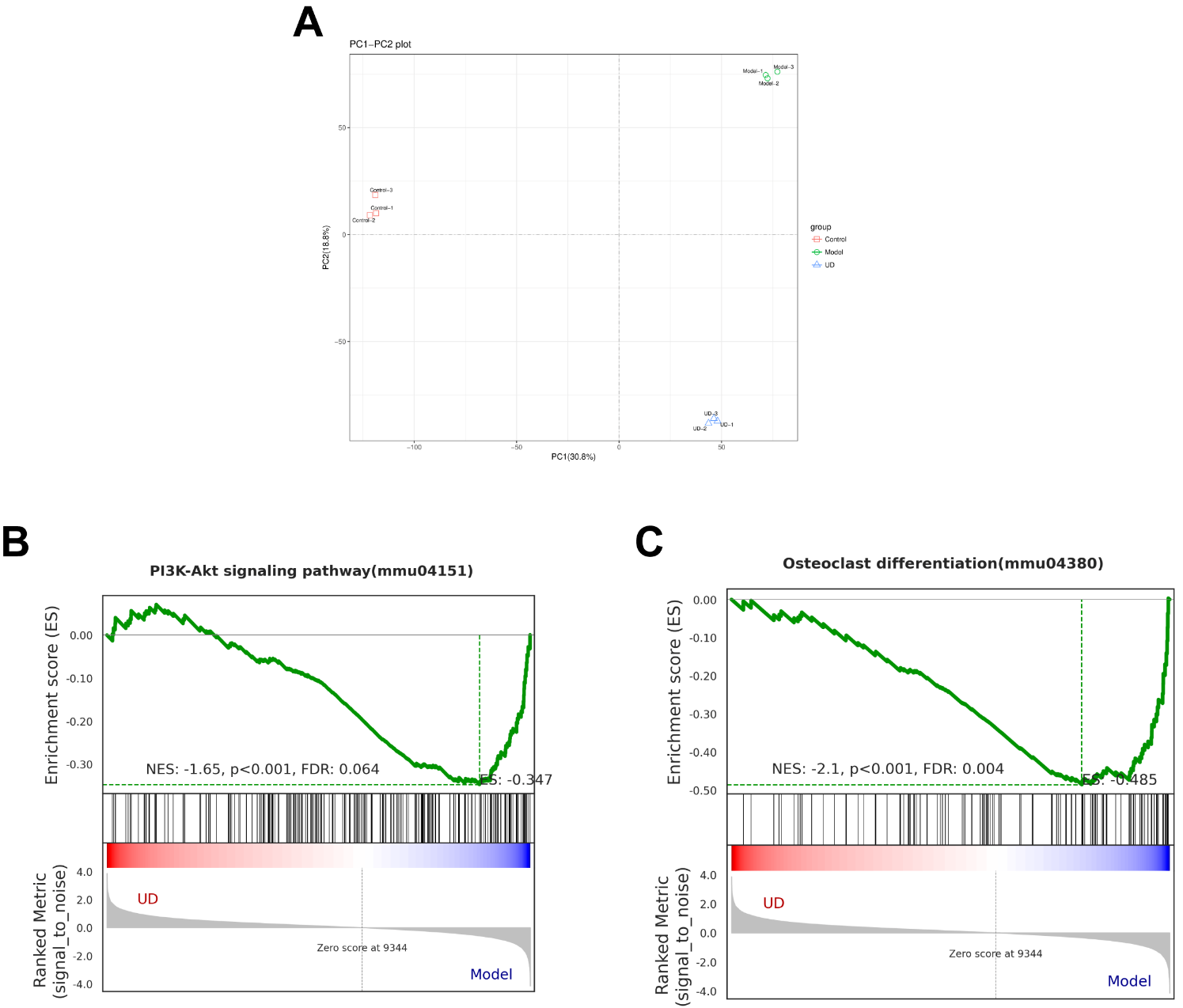


**Supplementary Figure S3. Transcriptomic analysis of RANKL-induced and UD-treated cells.**

(A) PCA of control, model, and UD groups; (B, C) GSEA of the PI3K–Akt signaling pathway and osteoclast differentiation pathway.


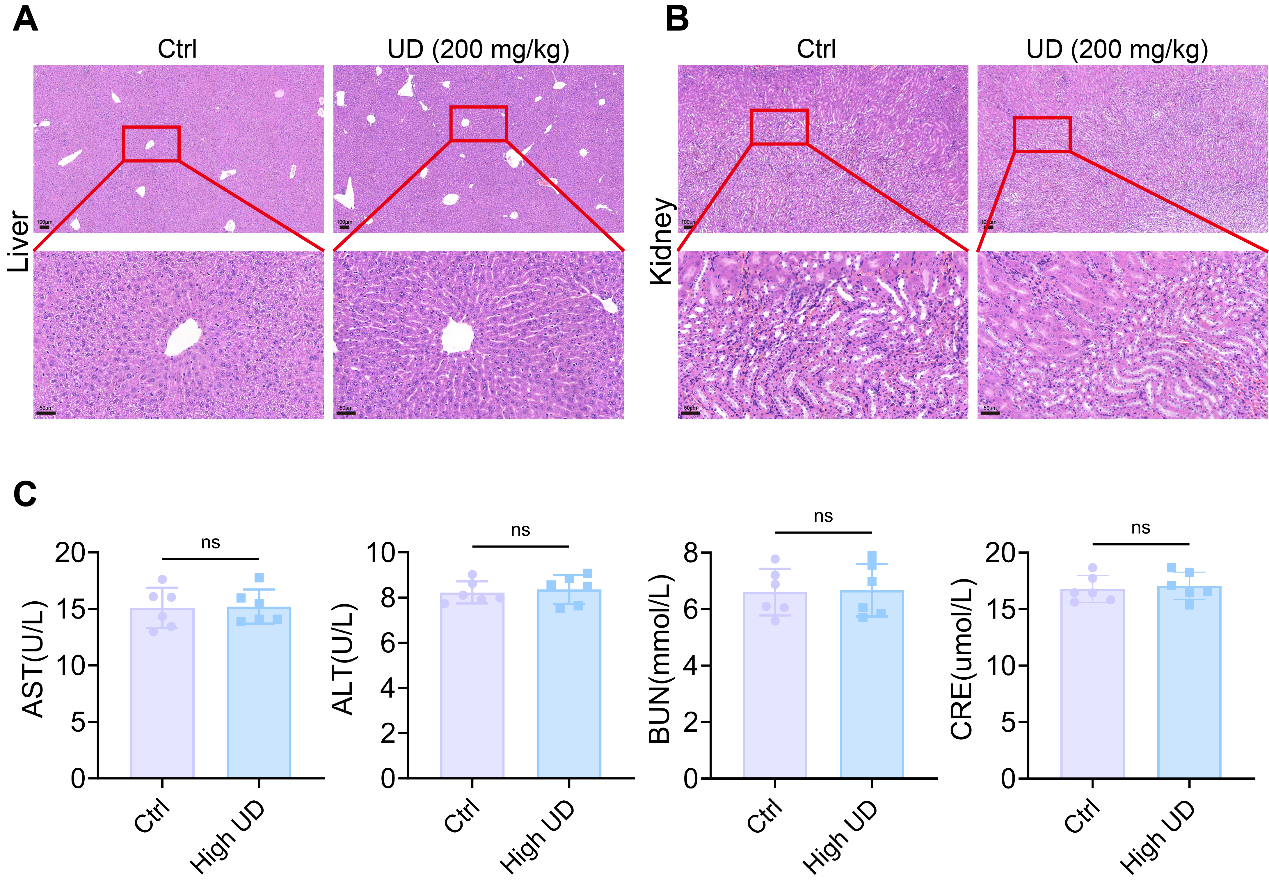


**Figure S4. Uridine does not induce liver or kidney toxicity**

(A) H&E staining of liver. (B) H&E staining of kidney. (C) Serum AST, ALT, BUN, and CRE levels. Data are presented as mean ± SD (n = 6). No significant differences were observed between groups (ns, P > 0.05).
