# Supplementary material for: Uridine inhibits ROS-mediated osteoclast differentiation and alleviates osteoporosis via modulation of PI3K/Akt–FoxO signaling
Source: Front Immunol. 2026 Apr 27;17:1767279. doi: 10.3389/fimmu.2026.1767279 (PMC13158086; doi:10.3389/fimmu.2026.1767279)

Raw data for Figure 2H – Western blot (uncropped)

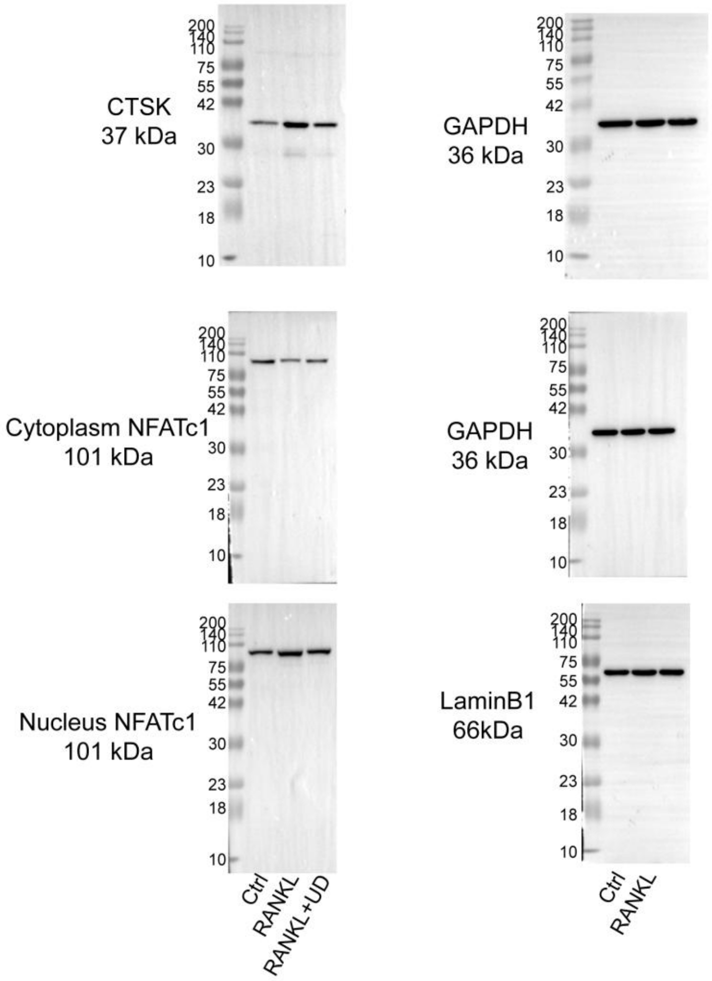

Raw data for Figure 3I – Western blot (uncropped)

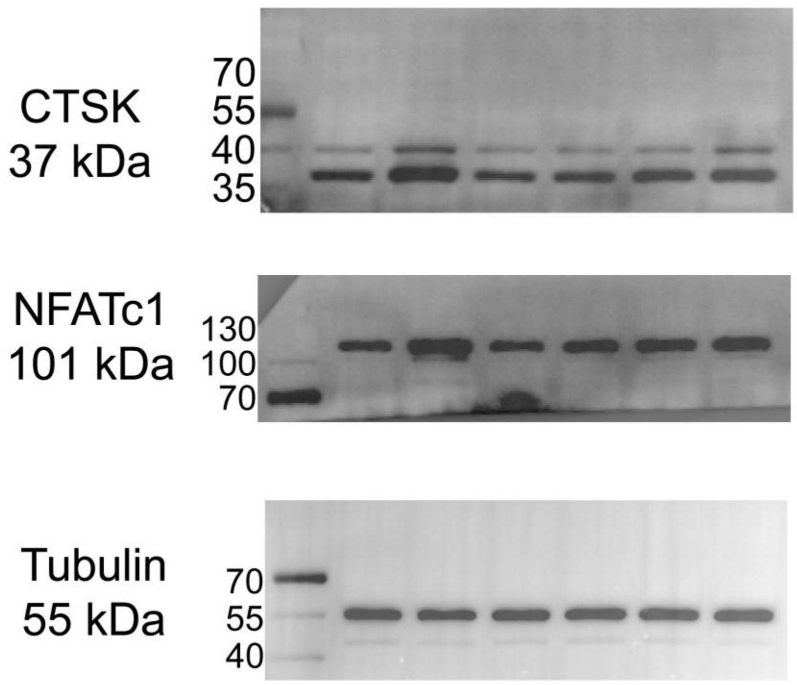

Raw data for Figure 4F – Western blot (uncropped)

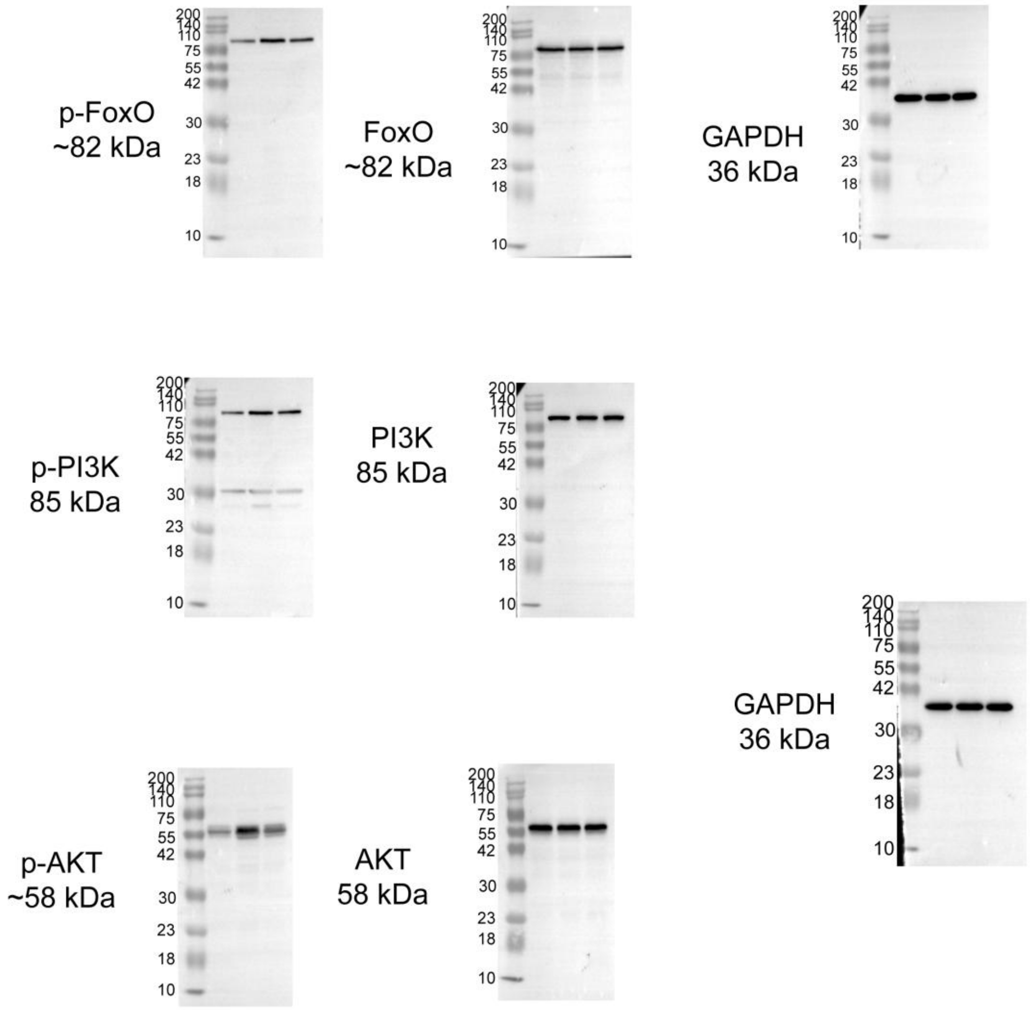

Raw data for Figure 4J – Western blot (uncropped)

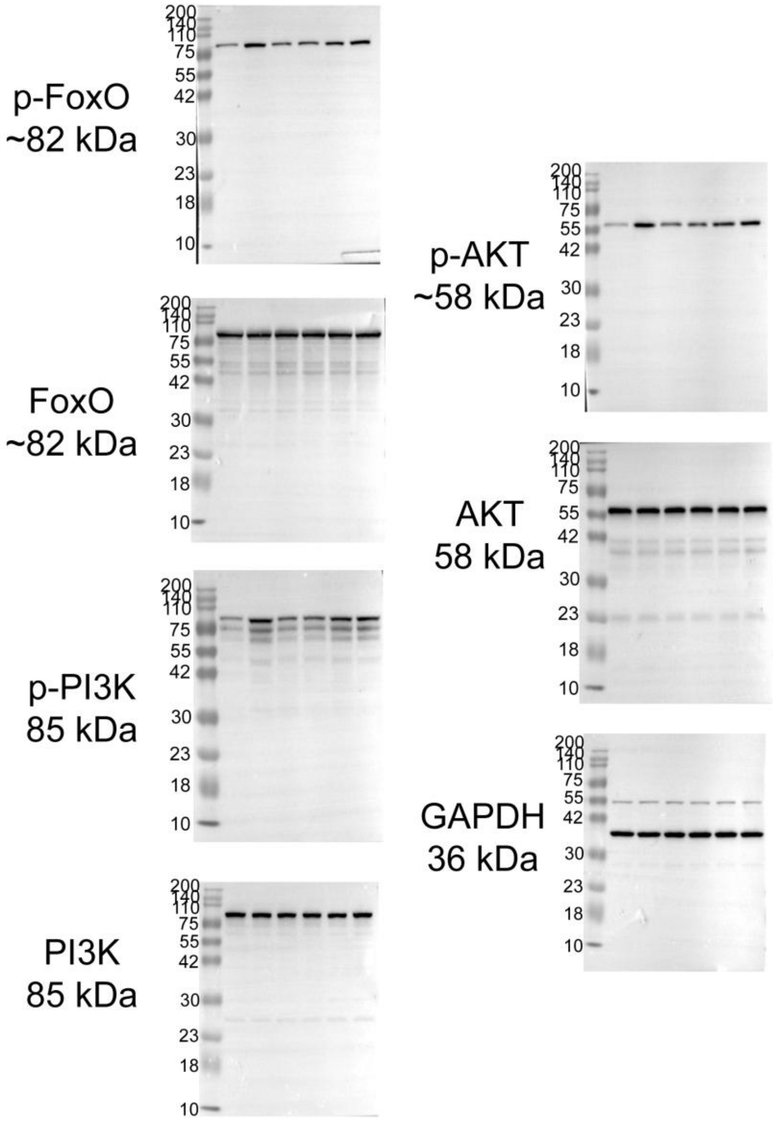

Raw data for Figure 5B – Western blot (uncropped)

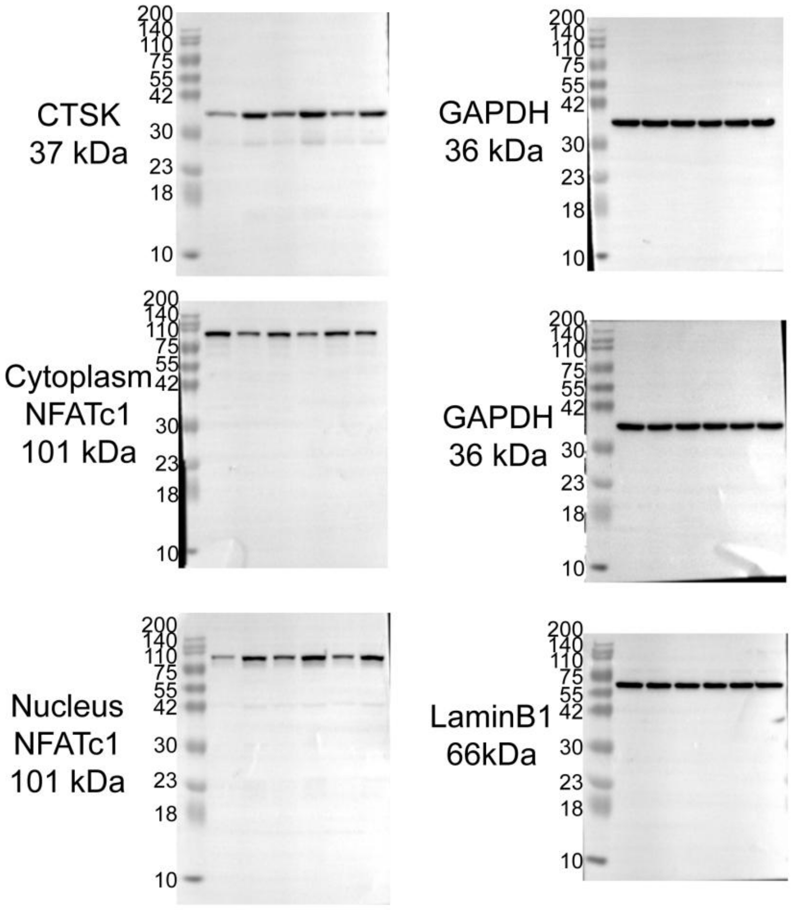

Raw data for Figure 6H – Western blot (uncropped)

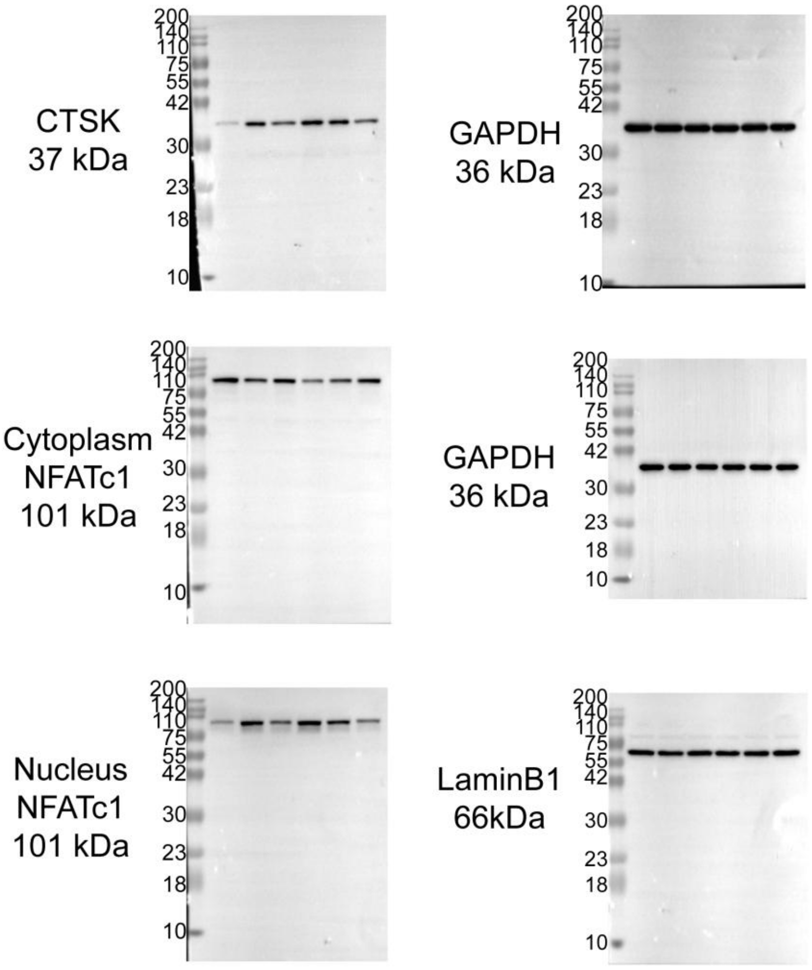

Raw data for Figure 6l – Western blot (uncropped)

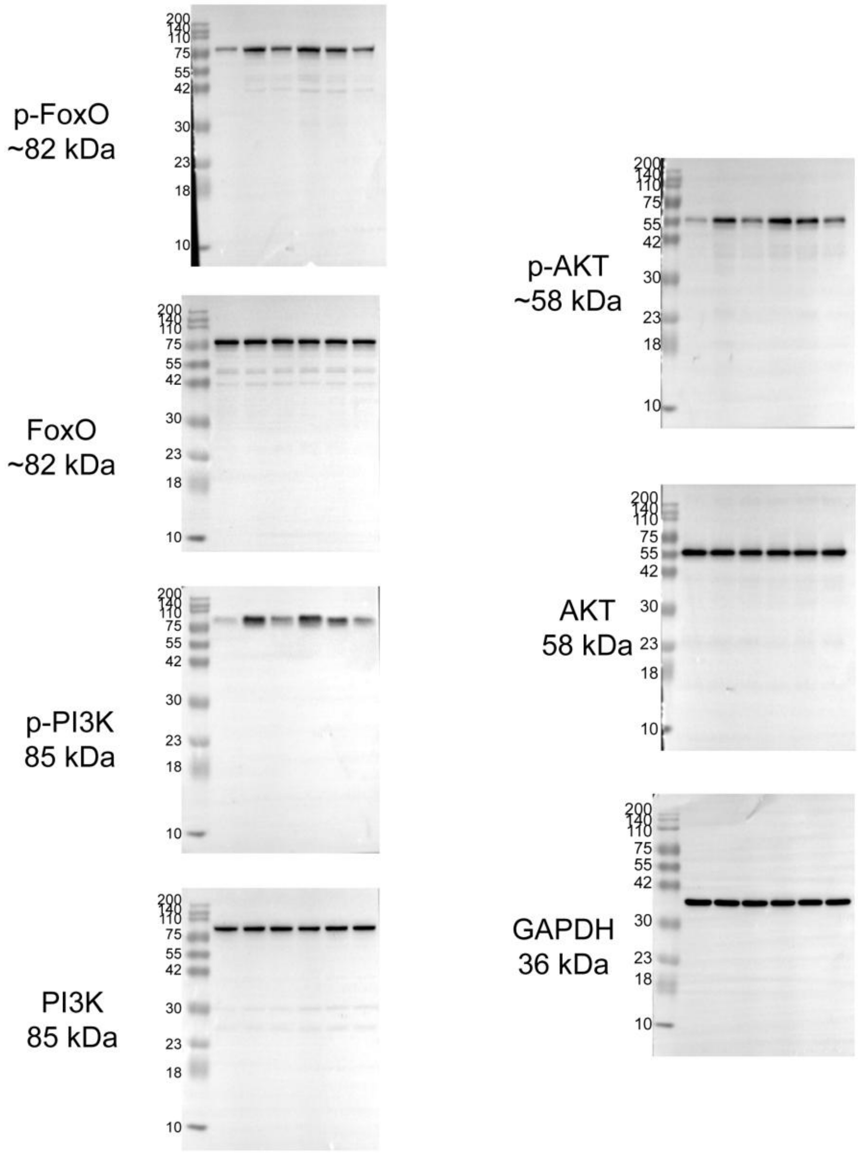

Supplement: Supplementary file 2 [file DataSheet2.pdf]
